# A revised diffusion model for conflict tasks

**DOI:** 10.3758/s13423-023-02288-0

**Published:** 2023-06-12

**Authors:** Ping-Shien Lee, David K. Sewell

**Affiliations:** School of Psychology, University of Queensland, QLD 4072 St. Lucia, Australia

**Keywords:** Cognitive control, Delta plots, Conflict tasks, Diffusion decision models, Attention shift

## Abstract

The recently developed diffusion model for conflict tasks (DMC) Ulrich et al. (*Cognitive Psychology*, *78*, 148–174, 2015) provides a good account of data from all standard conflict tasks (e.g., Stroop, Simon, and flanker tasks) within a common evidence accumulation framework. A central feature of DMC’s processing dynamics is that there is an initial phase of rapid accumulation of distractor evidence that is then selectively withdrawn from the decision mechanism as processing continues. We argue that this assumption is potentially troubling because it could be viewed as implying qualitative changes in the representation of distractor information over the time course of processing. These changes suggest more than simple inhibition or suppression of distractor information, as they involve evidence produced by distractor processing “changing sign” over time. In this article, we (a) develop a revised DMC (RDMC) whose dynamics operate strictly within the limits of inhibition/suppression (i.e., evidence strength can change monotonically, but cannot change sign); (b) demonstrate that RDMC can predict the full range of delta plots observed in the literature (i.e., both positive-going and negative-going); and (c) show that the model provides excellent fits to Simon and flanker data used to benchmark the original DMC at both the individual and group level. Our model provides a novel account of processing differences across Simon and flanker tasks. Specifically, that they differ in how distractor information is processed on congruent trials, rather than incongruent trials: congruent trials in the Simon task show relatively slow attention shifting away from distractor information (i.e., location) while complete and rapid attention shifting occurs in the flanker task. Our new model highlights the importance of considering dynamic interactions between top-down goals and bottom-up stimulus effects in conflict processing.

Daily life abounds with situations where cognitive control is needed to act on one cue at the expense of acting differently based on another cue. An ad implores you to indulge in a block of chocolate while the fitness app on your phone reminds you that there is still time to go for a run today. Investigating how people process such conflicting information is important for understanding goal-directed behavior, especially in rapid decision contexts where bottom-up stimulus inputs interact with top-down goals on a highly compressed timescale. For example, a driver approaching a green light while seeing an emergency stop from the car in front is a high-stakes real-world example where cues are associated with conflicting responses. The green light is strongly associated with continuing on driving, whereas the sudden stop from the car in front demands immediate braking to avoid a collision. When one of the cues has a processing advantage (e.g., due to automaticity or otherwise being highly practiced), cognitive control is required to rapidly shift processing resources and respond appropriately, in line with top-down goals.

To explain how cognitive control mechanisms influence conflict processing, theoretical models have posited dynamic changes in the processing of different stimulus components where these components are either congruent with one another (predicting a common response; no conflict) or incongruent (predicting different responses; conflict). Three major models that have been used in modelling conflict tasks are the shrinking spotlight model (SSP; White et al. , 2011), the dual-stage two-phase model (DSTP; Hübner et al. , 2010), and the diffusion model for conflict tasks (DMC; Ulrich et al. , 2015). Although these models differ in their scope (e.g., the SSP is a model of the flanker task in specific, whereas DMC and DSTP are general models of conflict tasks) and the precise ways in which they explain data from conflict tasks, they share the general assumption that conflict processing involves a dynamic transition from relatively non-selective processing (where both task-relevant and task-irrelevant stimulus components influence decision-making) to more narrowly selective processing (where decision-making is dominated by task-relevant stimulus components). Of these three models, DMC arguably has the widest range of applicability, as it can account for benchmark patterns of data (discussed below) that the SSP and DSTP cannot.

In this article, we critically re-examine some of the theoretical assumptions of DMC and argue that the processing assumptions implemented in the original model are potentially troubling because they can be interpreted as implying qualitative and non-monotonic changes in the psychological representation of stimulus information driving decision-making. These assumptions produce evidence accumulation dynamics that are consistent with a reversal in the distractor representation driving decision-making without any associated change in the stimulus (e.g., the same distractor representation initially supports response A, but later supports response B). Although such changes are sometimes explained in terms of inhibition (Tipper, 1985; Kane et al., 1997), we argue that allowing the representational changes implied by DMC’s processing dynamics goes beyond what inhibitory mechanisms are theoretically capable of. In this article, we develop a new revised diffusion model for conflict tasks (RDMC) that is based on a non-inhibitory framework that enforces strictly monotonic changes in stimulus representations over time (i.e., strengthening or weakening without any qualitative change in representations). RDMC achieves this by using a coupled activation function to dynamically control the relative influence of task-relevant and -irrelevant information over the course of an individual trial. Mechanistically, we interpret the activation function as reflecting the deployment of selective attention over task-relevant and task-irrelevant processing channels. We proceed by first providing a brief review of conflict tasks, summarizing key empirical benchmarks that motivated the development of DMC. We then review the theoretical assumptions underpinning DMC, highlighting the processing dynamics entailed by these assumptions. We then present a formal overview of our RDMC, showing the model has excellent parameter recovery properties. Finally, we show that RDMC provides excellent fits to the same benchmark data used to evaluate the original DMC (i.e., so-called positive- and negative-going delta plots), providing a novel account of the divergent characteristics of data from Simon and flanker tasks. We further show through simulations that the same processing dynamics that allow our model to account for negative-going delta plots are also sufficient to produce negative congruency effects conclude by discussing future research directions that could provide further tests of RDMC.

## Review of conflict tasks

Three canonical tasks that have been used to study conflict information processing are the Stroop, Simon, and flanker tasks. In these tasks, participants make speeded responses to a target while ignoring task-irrelevant distractor information. Trials vary in terms of whether the target and distractor information are associated with a common response (congruent trials) or with different responses (incongruent trials). Responses on congruent trials are generally faster than responses on incongruent trials. For example, in the Stroop task (MacLeod, 1991; Stroop, 1935), people are shown a word presented in colored ink, and are required to name the ink color (target information) while ignoring the word meaning (distractor information). People tend to respond slower and are more error-prone when presented with an incongruent stimulus (e.g., RED displayed in blue ink) than with a congruent stimulus (e.g., RED displayed in red ink). The response time (RT) difference between congruent and incongruent trials is known as the *congruency effect*. Simon and Rudell (1967) later introduced the Simon task in which people respond to the auditory presentation of the word “left” or “right” (target information) by pressing a corresponding button while ignoring “location” information (whether the word was presented to the left or right ear; distractor information). Lastly, in the classic flanker task by Eriksen and Eriksen (1974), people respond to a target letter flanked by distractor letters that are either the same as, or different from, the target letter.^1^

The magnitude of the congruency effect is interpreted as reflecting the time course of enacting cognitive control. Under some accounts, this involves shifting attentional resources away from distractor information to focus on the target. Under other accounts, distractor information is actively suppressed by inhibitory mechanisms. The larger the congruency effect, the longer it takes to adjust ongoing processing dynamics (e.g., through inhibition or reallocation of attentional resources). Theoretical treatments of cognitive control are often based around two channels of information processing, automatic and controlled (Schneider and Shiffrin, 1977; Shiffrin and Schneider, 1977). Within this framework, conflict processing is often explained in terms of dynamically adjusting the pool of resources supporting target information processing carried by the controlled channel vs. distractor information processing carried by the automatic channel. For example, the shrinking spotlight model (SSP) proposed by White et al. (2011) combines the idea of visual attention and cognitive control. The model narrows an attentional spotlight to effectively prevent distractor information from continuing to influence decision-making. The size of the congruency effect is determined, in part, by how quickly the attentional spotlight is narrowed, and consequently, how rapidly attentional resources become concentrated on the target. Alternatively, inhibition-based accounts envisage inhibitory connections across processing channels with the size of the congruency effect reflecting the time course of inhibiting processing in the automatic channel.

Congruency effects are universal across conflict tasks and are typically positive, reflecting slower average RTs for incongruent trials.^2^ In terms of automatic and controlled processing, congruency effects describe whether automatic processing facilitates or interferes with target processing. For congruent trials, distractor information can potentially facilitate target processing since all stimuli are associated with the same correct response. If target and distractor information is pooled across automatic and controlled channels, RTs on congruent trials should be faster. For incongruent trials, distractor processing creates interference because distractors are associated with the opposite incorrect response to the target. Pooling information across automatic and controlled channels in this case results in slower RTs as distractor evidence negates target evidence.

### Time dynamics of congruency effects

Notwithstanding the similarities between congruency effects produced by different tasks at the level of mean RTs, there are other more subtle patterns in congruency effects that differ reliably across conflict tasks (Pratte et al., 2010). When conditioned on overall RT, there are pronounced differences in the magnitude of congruency effects over time with effects sometimes growing and sometimes shrinking over the course of a trial. Congruency effects tend to increase with time in flanker tasks, but tend to collapse over time in Simon tasks. A major theoretical challenge has been to characterize these different dynamic patterns of congruency effects. The time-varying strength of congruency effects can be visualized using so-called delta plots, which show RT differences between congruent and incongruent stimuli at different points in time (De Jong et al., 1994). Congruency effects often vary across the RT distribution, so delta plots can be characterized by their positive or negative slopes. Positive-going delta plots are found consistently in flanker tasks while negative-going delta plots are usually shown in Simon tasks (Pratte et al., 2010; Ridderinkhof et al., 2005). Differences in delta plots across conflict tasks have been important with respect to theory, as they may reflect different mechanisms underlying conflict processing in different tasks (Burle et al., 2014; Pratte, 2021; Ridderinkhof, 2002a, b). However, there is a wide range of mechanisms that can produce positive- and negative-going delta plots, and so formal modeling is required to contrast different assumptions.

Delta plots also provide an additional means of quantitatively assessing the adequacy of theoretical models of conflict effects. In addition to fitting congruency effect data at the level of RT distributions for different tasks, successful models must also be able to account for differences in the magnitudes of congruency effects over time. Positive-going delta plots are commonly predicted by major conflict diffusion models (e.g., DSTP; Hübner et al., 2010, SSP; White et al., 2011, DMC; Ulrich et al., 2015). Conversely, negative-going delta plots cannot be produced by most conflict diffusion models including SSP and DSTP (Servant et al., 2014). Indeed, Pratte et al. (2010) argued that negative-going delta plots could not readily be accommodated by standard evidence accumulation models. However, Schwarz and Miller (2012) demonstrate that several models including standard diffusion models can account for both positive- and negative-going delta plots under certain specific assumptions.^3^ It follows that formal modeling is required to determine whether the assumptions of a given model allow it to produce negative-going delta plots. Of the three major models of conflict processing, only DMC has been shown to model both positive- and negative-going delta plots (Ulrich et al., 2015). The success of DMC has been attributed to dynamic changes in the way distractor information is accumulated over the course of a trial, producing symmetrical facilitation and interference effects on congruent and incongruent trials, respectively. An extension of DMC that allows for asymmetrical facilitation and interference effects was examined recently by Evans and Servant (2022). The processing dynamics within the model are such that the time course of evidence accumulation by distractor information can produce both positive- and negative-going delta plots, making DMC an attractive model for studying processing across a range of conflict tasks.

In addition to delta plots, accuracy rates across RT distributions are also important benchmarks for evaluating models of conflict processing. Conditional accuracy functions (CAFs) plot accuracy as a function of response speed for congruent and incongruent trials (Heitz, 2014). Congruent conditions tend to obtain higher and more consistent accuracy levels across the entire RT distribution, while incongruent conditions tend to have a higher error rate for fast responses, and a comparable accuracy rate to congruent conditions for slower responses (e.g., Ridderinkhof , 2002a; Stins et al. , 2007). The patterns of CAFs do not vary much across different conflict tasks, and are accurately fitted by most models.

Past literature has highlighted the importance of RT distribution data, the corresponding delta plots, and the CAFs (Pratte et al., 2010; Schwarz and Miller, 2012; Heitz, 2014). These benchmarks are able to distinguish distinct RT patterns in different conflict tasks and provide more detail than the averaged RT or accuracy data for congruent vs. incongruent trials. Any successful general model of conflict processing must account for all of the benchmarks. At present, DMC is the one existing model that has been shown to accommodate these benchmarks in both Simon and flanker tasks (Ulrich et al., 2015). We now review the formal properties of DMC.

## Revisiting DMC

DMC is based on the idea of automaticity, which has been influential in the cognitive control literature (Schneider and Shiffrin, 1977; Shiffrin and Schneider, 1977). Within this framework, stimuli are conceptualized as comprising both task-relevant and task-irrelevant features. DMC assumes that responses are driven by a combination of task-irrelevant and task-relevant information accumulated by automatic and controlled processing channels respectively, but the impact of these two channels on decision-making varies over time. For example, in the flanker task, the task-irrelevant information of the flanker arrows is accumulated in the automatic channel and the task-relevant information of the target arrow is accumulated in the controlled channel. It follows that the automatic process accumulates distractor information supporting correct/incorrect responses on congruent/incongruent trials, and the controlled process accumulates target information that always supports a correct response. Information from the two evidence channels is combined into a single evidence total. A response is initiated once the combined evidence reaches one of two response boundaries, corresponding with the two response alternatives (e.g., a left- or right-pointing target in the flanker task).

**Fig. 1:**
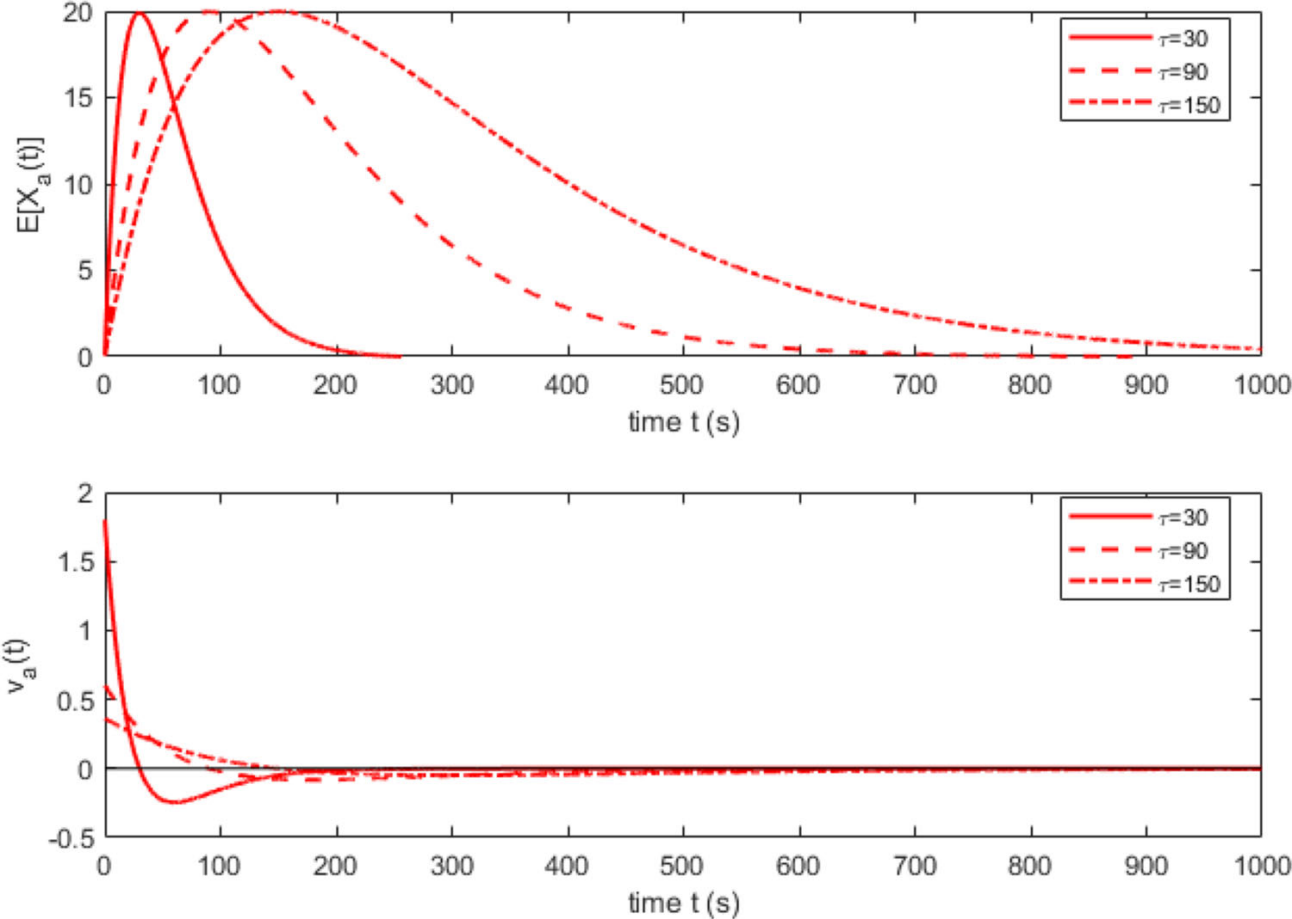
Expected automatic evidence accumulation trajectories (*upper panel*) and their corresponding automatic drift rates (*lower panel*) for three different values of $\tau$. Larger values of $\tau$ correspond to slower dynamics in the automatic channel. The scaling parameter for the pulse function, *A*, is fixed at 20 and the shape parameter of the gamma distribution $\alpha$ is fixed at 2. Because the automatic drift rate is the first derivative of the expected evidence total, the drift rate changes signs partway through the evidence accumulation process before converging to 0. Figure adapted from Ulrich et al. (2015)

A core concept in DMC is the dynamic influence of evidence accumulated by the automatic channel. Early on in processing, before attention can effectively filter out task-irrelevant information (or inhibitory mechanisms suppress automatic processing), evidence accumulation is dominated by the automatic channel. Later in processing, when attention is focused more narrowly on task-relevant components of the stimulus (or after automatic processing has been inhibited), evidence accumulation is dominated by the controlled channel. DMC models the time course of these processing dynamics in terms of the expected amount of evidence accumulated by the automatic channel through time, $E[X_a(t)]$, using a pulse function that rises to a maximum value before decreasing back to a baseline of zero. Within DMC, the late withdrawal of evidence previously accumulated by the automatic channel is attributed to inhibition of the automatic channel (Hommel, 1994; Lu and Proctor, 2001). Formally, these dynamics are modeled using a rescaled gamma density function,

1 $$\begin{aligned} E[X_a (t)]=Ae^{-t/\tau }\left[ \frac{te}{(\alpha -1)\tau }\right] ^{\alpha -1} \end{aligned}$$

with a fixed shape parameter $\alpha$ > 1, a free scale parameter $\tau$, and multiplied by a free scaling parameter *A*. Equation 1 describes the output of a diffusive evidence accumulation process with time-varying drift rate $v_a(t)$, obtained by taking the first derivative of $E[X_a(t)]$ with respect to time, *t*. As with the standard diffusion model (Ratcliff, 1978; Ratcliff and McKoon, 2008), drift rates in DMC reflect the quality of information entering the decision process. The drift rate for the automatic channel, $v_a(t)$, represents the quality of distractor information present in the stimulus and available to the decision mechanism at time *t*. The time-varying nature of the automatic drift rate arises due to attentional filtering dynamically restricting task-irrelevant information from entering the decision process (or alternatively, from active inhibition of the automatic channel). The top panel of Fig. 1 shows the expected automatic evidence accumulation, $E[X_a(t)]$ for three different values of $\tau$. The bottom panel of the figure shows the corresponding automatic drift rates, $v_a(t)$.

The dynamics of the controlled channel are simpler, as evidence is expected to accumulate as a linear function of time, $E[X_c(t)]=v_ct$, yielding a constant controlled drift rate equal to $v_c$. Decisions in DMC are determined by the combined evidence accumulated by the automatic and controlled channels. As noted by Ulrich et al. (2015), evidence accumulation can be modeled as a single combined Wiener process

2 $$\begin{aligned} dX(t)=\left[ v_a(t)+v_c\right] dt+\sigma dW(t) \end{aligned}$$

where $\sigma$ is the square root of the diffusion coefficient, and *W*(*t*) is a white noise process (i.e., a sample from a Gaussian distribution with mean 0 and variance 1). $\sigma$ is typically fixed to an arbitrary constant, serving as a scaling parameter^4^ (see Donkin et al. (2009), for critical discussion of this assumption). Evidence accumulation for the combined process begins at a starting point, *z*, and continues until the process hits one of two absorbing boundaries, located at 0 and *a*, initiating the corresponding response. The automatic process accumulates evidence toward the upper boundary located at *a* (i.e., correct responses) on congruent conditions, and toward the boundary located at 0 (i.e., incorrect responses) on incongruent conditions. The controlled process always accumulates evidence toward the correct response boundary located at *a*.

**Fig. 2:**
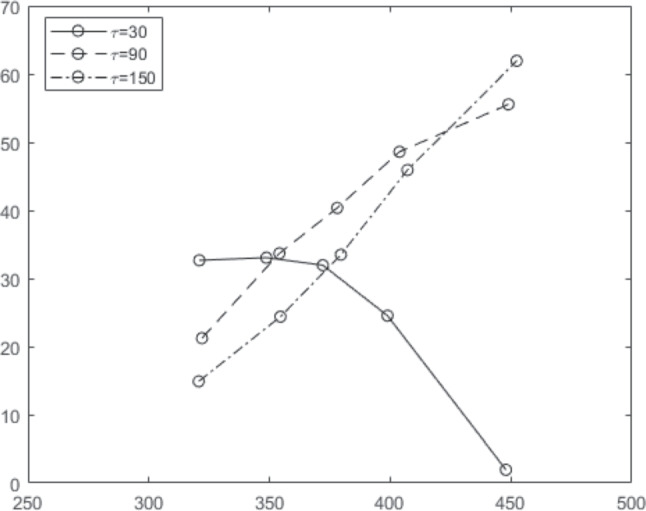
Delta functions produced by DMC based on the dynamics illustrated in Fig. 1. The functions express the response time difference for the 0.1, 0.3, 0.5, 0.7, and 0.9 distribution quantiles for correct responses on congruent versus incongruent trials. The slope of the delta functions differ according to the values of $\tau$. The controlled drift $v_c$ is fixed at 0.7, the boundary separation *a* is fixed at 100, and the diffusion coefficient is fixed at 3. Finally, the non-decision time is given by a bounded uniform distribution with a mean 310 and a range 80

The dynamics of the automatic channel in DMC allow a continuous transition between predicting positive- and negative-going delta plots. Slopes of the delta plots produced by the model are determined by the magnitude of the scale parameter $\tau$ in Eq. 1 with negative-going delta plots associated with smaller values of $\tau$ (Ulrich et al., 2015). This suggests that negative-going delta plots arise when automatic channel activation has a fast time course, characterized by a strong initial response that is rapidly reduced back to zero. Delta plots produced by the model at the same three values of $\tau$ from Fig. 1 are shown in Fig. 2. Higher values of $\tau$ are associated with steeper positive-going delta plots. When $\tau =30$, the delta plot produced by the model is negative-going.

A distinct advantage of DMC over other models is its ability to qualitatively account for both positive- and negative-going delta plots (Servant et al., 2014). Importantly, several studies demonstrate that DMC also successfully provides good quantitative fits to joint accuracy and RT distribution data from both Simon and flanker tasks, including cases involving negative-going delta plots (Ellinghaus et al., 2018; Hübner and Töbel, 2019; Servant et al., 2016; Ulrich et al., 2015). Moreover, DMC has been implemented in the Simon task with electrophysiological measurement supporting the early development of processing in the automatic channel (Servant et al., 2016).

## Critique of DMC

In this article, we critically revisit DMC’s assumptions about evidence accumulation in the automatic channel. We argue that the model’s assumptions lead to potentially troubling implications for how distractor information is represented over the course of a trial. In particular, the use of a pulse function to describe the cumulative output of the automatic channel (i.e., total evidence accumulated through time) enforces an evidence accumulation process involving rapid initial accrual of evidence based on distractor processing followed by the gradual withdrawal of that evidence from the decision process.^5^ While the theoretical intent of the pulse function is to describe the short-lived dominance of the automatic channel over the decision process, in practice, the function implies changes in both the sign and the strength of the representations that drive evidence accumulation based on the automatic processing channel in the absence of any changes in the stimulus. That is, DMC’s pulse function decouples encoded properties of the stimulus from model parameters that govern the evidence accumulation process (i.e., drift rates). Given the central role of the pulse function in accounting for the variety of delta plots found empirically (Ulrich et al., 2015), it is important to consider whether the same patterns of results can be explained by different processing assumptions that enforce consistent representation of fixed stimulus properties. Our main theoretical contribution in this article is to show that an alternative implementation of the automatic and controlled processing framework that underpins DMC successfully accounts for key benchmark effects—including negative-going delta plots—in a way that avoids unexplored and potentially problematic theoretical implications introduced by the pulse function (Eq. 1).

In DMC, the drift rate describing evidence accumulation in the automatic channel is time-varying, reflecting dynamic changes in the availability of task-irrelevant information to the decision mechanism described by Eq. 2. This view of automatic drift rates in DMC aligns with the conventional interpretation of drift rates in the standard diffusion model, as summarizing the quality of stimulus information (Ratcliff and McKoon, 2008). Identifying drift rates with quality of evidence provided by the stimulus accords with the multitude of previous studies that have found drift rates to vary systematically as a function of the signal-to-noise ratio of the stimulus (e.g., in dot motion tasks; Ratcliff and McKoon , 2008;Palmer et al. , 2005). Drift rates are also sensitive to manipulations that affect the ease with which stimuli with maximally different features are perceived (e.g., contrast manipulations with orthogonal stimuli; Sewell and Smith 2012; Smith et al. 2010, 2004). Moreover, stimulus-specific drift rates that track associative relationships of varying strength can be learned through trial-by-trial feedback (e.g., Fontanesi et al. 2019; Miletić et al. 2021; Pedersen et al. 2017; Sewell et al. 2019; Sewell and Stallman 2020). To the extent that the encoded properties of a stimulus remain consistent,^6^ during processing, we argue that dynamic changes in drift rates must be limited to varying only in strength (i.e., allowing quantitative changes in the magnitude of the drift rate, but not qualitative changes in the sign of the drift rate).

Formally, the decoupling of fixed stimulus properties from automatic drift rates can be observed by noting that the derivative of the function describing the expected value of cumulative evidence over time yields the time-dependent drift rate of the process that generated the evidence. The form of the pulse function is such that the initial steep rise in cumulative evidence entails a positive-valued drift rate on a congruent trial. The pulse function then reaches an inflection point, after which there is a withdrawal of previously accumulated evidence as the function returns to zero. The latter dynamics entail a sign change in the drift rate before and after the inflection point. Eventually, the drift rate for the automatic processing channel stabilizes at zero as decision-making becomes governed exclusively by information carried by the controlled processing channel.

One way to potentially reconcile DMC’s pulse function with theory is to assume that the mechanism responsible for the pulse is evidence leakage (as in Usher and McClelland (2001)) or spontaneous decay of information from the automatic channel (Hommel, 1994; Lu and Proctor, 2001). There are arguments that militate against this stance, as support for leakage assumptions is mixed. Ratcliff and Smith (2004) found that in fits to behavioral data, leakage estimates typically converged to 0 (resulting in similar behavior to the standard Wiener diffusion model). Other studies using time-varying stimuli similarly found no support for leakage (Evans et al., 2017). A later study involving a more comprehensive set of model comparisons found some support for leakage operating on a timescale of 200-250 ms (Trueblood et al., 2021), though we note that the timescale seems at odds with the rapid time course required to produce negative-going delta plots (e.g., corresponding to $\tau =30$ in Figs. 1 and 2). Moreover, reversal of the sign of the automatic drift rate requires rapid onset of leakage that greatly outpaces the rate of evidence accumulation for the automatic process. Under this view, the effect of leakage would also appear to be asymmetrical, affecting the controlled process to a much lesser extent. Overall, interpreting the dynamics of the pulse function in terms of evidence leakage seems not especially viable.

**Fig. 3:**
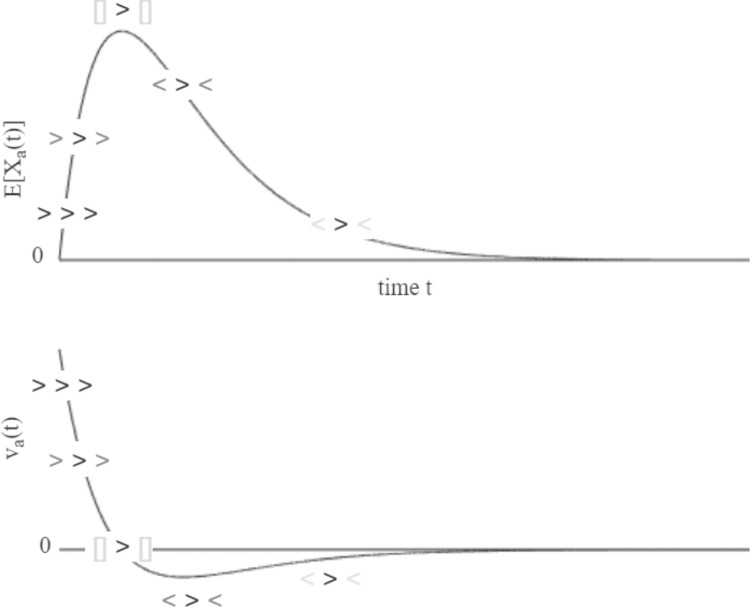
Representation of flanker arrows implied by time-dependent changes in automatic drift rate in DMC. If drift rates are viewed as reflecting encoded properties of the stimulus, it is difficult to reconcile how fixed stimulus properties give rise to representations that vary qualitatively over time. A key point of contention is that the sign change of the automatic drift rate would seem to imply a reversal of the information encoded by distractor stimuli

If, alternatively, the pulse function is interpreted as an after-the-fact withdrawal of evidence from the decision mechanism that is driven by inhibition, we argue that this interpretation is mechanistically fraught. To our minds, in regard to conflict tasks, the purpose of inhibition is to suppress or prevent ongoing processing of non-target information. Logically, such a mechanism suffices to change the drift rate of the automatic process from a non-zero value to zero, reflecting the cessation of further processing. To explain the sign-change entailed by DMC’s pulse function, inhibition must go further and unring the bell of previously accumulated distractor information, by selectively accumulating “counter-evidence” that is contrary to the properties of the distractor stimuli^7^ (See Fig. 3 for an example of a single flanker trial). It is the necessity of reversing the outcome of previous processing—rather than simply halting continuing processing—that is entailed by DMC’s pulse function that motivates us to establish whether it is possible to provide a general account of conflict data (and especially for negative-going delta plots) through some other processing assumptions. Further, we ask to what extent DMC’s past successes rely on the later withdrawal or negation of previously accumulated evidence. We are not aware of any mechanism other than selective inhibition that would enable such sophisticated tracking of evidence samples through time. If it is possible to account for the range of benchmark data that supported DMC using a simpler set of processing assumptions (i.e., avoiding the withdrawal of distractor evidence and the capacity for inhibition to reverse—rather than simply halt—ongoing evidence accumulation dynamics), we believe it is preferable to do so.^8^

**Fig. 4:**
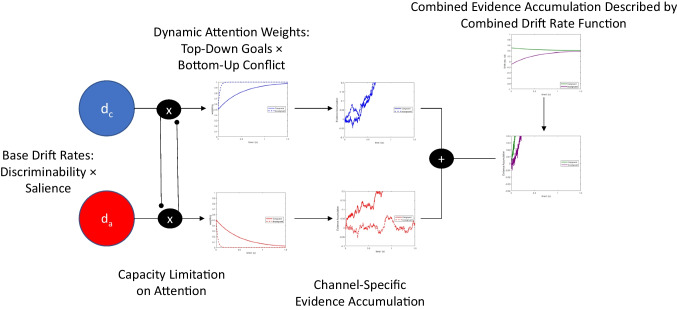
RDMC model components and interaction dynamics. Base drift rates $d_c$ and $d_a$ represent the properties of target and distractor stimuli, respectively. We assume base drift rates depend on the relative salience of target and distractor stimulus elements. The influence of target and distractor information on decision-making is dynamically modulated in RDMC via time-dependent attention weights that reflect the interaction of top-down goals with bottom-up characteristics of the stimulus display. Mathematically, the channel-specific activation is the product of the time-dependent attention weight and base drift rate. As in the original DMC, evidence accumulation is driven by the combined drift rate that is based on automatic and controlled channel activation

## The revised diffusion model for conflict tasks (RDMC)

We propose a revised DMC (RDMC) based on the original DMC model framework of automatic and controlled processing (i.e., short-lived automatic processing alongside sustained controlled processing). Our framework enforces consistency in the way task-irrelevant information is represented over the course of processing by relying on dynamic normalization of the strength of automatic channel activation over time (Sewell et al., 2014; Smith and Sewell, 2013; Smith et al., 2015). We show that RDMC successfully fits RT distribution data from both flanker and Simon tasks, naturally generating their corresponding positive- and negative-going delta plots without requiring inhibition or changes in how distractor information is represented over time. That is, we show that conflict data can be explained without requiring a mechanism that can reverse the sign of evidence previously accumulated by the automatic channel. We fit the model to the aggregated Simon and flanker data from Ulrich et al. (2015), showing our revised model produces visually comparable fits to the data than the original DMC. We present RDMC as a non-inhibitory account of conflict tasks that has the potential to be tested against alternative inhibition accounts directly. We therefore show that negative-going delta plots in particular can be explained within a purely feed-forward modeling framework and that late withdrawal of previously accumulated evidence is not needed to explain the variety of delta plots that are observed in the literature.

In RDMC, we assume that the output of automatic and controlled channels cannot be altered once it has entered the decision process (i.e., initial evidence generated by the automatic channel cannot be withdrawn later in processing). Consequently, evidence outputted by the automatic channel persists, but becomes relatively less influential over time in the face of continuing evidence output produced by the controlled channel (see Fig. 4 for the architecture of RDMC). To model the flow of information outputted by the two channels over time, we allow the drift rates for both automatic and controlled processes to vary over time, using a normalized weighting function that jointly determines the activation levels of the two channels. Specifically, we model changes in automatic channel activation weight using an exponential decay function,

3 $$\begin{aligned} w_a(t) = A_{0}e^{-kt} \end{aligned}$$

and set controlled channel activation to

4 $$\begin{aligned} w_c(t) = 1 - w_a(t) \end{aligned}$$

In Eqs. 3 and 4, the initial activation level of the automatic channel (i.e., at $t=0$) is given by $A_{0}$, which is bounded within the interval [0,1].^9^ The value of $A_{0}$ reflects the observer’s initial distribution of processing resources across automatic and controlled channels. The rate at which attentional resources are diverted from the automatic channel to the controlled channel is given by *k*. Based on both empirical precedent (Craft and Simon, 1970; Eriksen and Eriksen, 1974; Simon and Small, 1969) and recent modeling work by Evans and Servant (2022), we allow the attention shift parameter, *k*, to differ for congruent and incongruent trials, reflecting potential differences in facilitation and interference effects observed in Simon and flanker tasks. We denote the attention shift parameter on congruent trials as $k_c$ and on incongruent trials as $k_i$. The potential for asymmetric facilitation and interference—or asymmetrical enacting of cognitive control more broadly—follows logically from the demands of conflict tasks. Responding correctly to incongruent trials requires filtering out distractor information. By contrast, distractor filtering is not necessary on congruent trials, though people may continue to do so owing to larger strategic considerations. We argue that differences in the speed of attention shifting reflect task-dependent differences in the way top-down goals interact with bottom-up stimulus information.

The coupled functions in Eqs. 3 and 4 describe dynamic changes in the way attention is allocated across the two processing channels, reflecting the operation of cognitive control. Kinder et al. (2022) used mouse tracking to provide recent evidence supporting continuous attentional selection in the flanker task, consistent with our assumption of smooth continuous changes in channel activation. The attention dynamics illustrate the interplay between top-down and bottom-up factors, as exemplified by Folk and Remington ’s (1998) work on attention capture. At the task level, there is a constant top-down goal of identifying the target. At the trial level, there is bottom-up information about the congruency of stimulus elements that is variable across trials. The top-down goal always provides impetus to shift attention onto the target. Bottom-up congruency information determines the urgency with which the shift must occur in order to achieve the goal. Shifting attention is necessary on incongruent trials and must occur rapidly to minimize the chance of an error. On congruent trials, however, target identification can be achieved regardless of how quickly attention is focused on the target (Luo and Proctor, 2016; Moore et al., 2021). Our assumption is that congruency modulates the speed with which attention is shifted in pursuit of the top-down goal of identifying the target. We consider trial congruency as a high-order emergent feature of the stimulus configuration that is based on the homogeneity of stimulus elements (e.g., as in flanker tasks) or the conceptual alignment of elements (e.g., as in Stroop and Simon tasks). Importantly, assessments of congruency are independent of identification of the target or distractor elements, and so the detection of conflicting elements or alignment does not rest on knowledge of the target. Mechanistically, we envisage a conflict detection mechanism that monitors activation levels of nodes that code for different stimulus properties (e.g., Yeung et al. , 2004). Conflict detection occurs when multiple nodes are activated above some threshold to filter background activation levels. Critically, the mechanism requires no awareness of the properties the nodes code for, just their activation levels. The influence of this bottom-up conflict signal on attention shifting depends on the strength of the conflict signal relative to the activation strength of a top-down goal representation.^10^ Following the framework of Botvinick et al. (2004), conflict detection is a specific role of anterior cingulate cortex (ACC) that should occur before the process of conflict resolution which is mostly implemented in the dorsolateral prefrontal cortex (DLPFC; Miller and Cohen , 2001; Carter and van Veen , 2007). Botvinick et al. ’s 2004 idea can be adapted to general conflict detection and resolution such as Stroop, flanker, and Simon tasks.^11^ Indirect support for the notion of rapid conflict detection comes from several sources. In visual perception and texture segmentation, global features are known to take precedence over local features (Navon , 1977; for a review, see Hegdé , 2008). Visual pattern recognition has been shown to be highly efficient (Bergen and Julesz , 1983), occurring within 100 ms, and has classically been relied upon to explain highly efficient visual search (Treisman and Gelade, 1980). While these pattern recognition results are perhaps most relevant to the flanker task, other examples of congruency-related factors affecting processing may be more relevant to the Simon and Stroop tasks. For example, the McGurk effect arguably demonstrates rapid congruency-related influences on perceptual processing (McGurk and Macdonald, 1976). More generally, conceptual priming effects on lower-level perceptual processing show how the match between a conceptual representation and stimulus information can rapidly modulate ongoing processing (e.g., Lupyan and Ward 2013; Oliva and Torralba 2007). Further evidence from Ariga and Yokosawa (2008) suggests that congruency detection can also be evaluated at a more abstract level of representation in which a distractor shares an abstract semantic level of representation with a target. We therefore argue that the idea of conflict detection also applies at the level of response conflict arising in Simon tasks and at the level of semantic meaning in Stroop tasks, not only at perceptual in flanker tasks. More generally, conflict detection can occur in an absence of attention/awareness (Xiang et al., 2013; Nuiten et al., 2021), and its rapid time course is supported by ERP evidence (Larson et al. , 2014 for review, Kałamała et al. , 2018, Ghin et al. , 2022, Coderre et al. , 2011). To summarize, Eqs. 3 and 4 characterize the time course of attention shifting in a way that captures the dynamic interplay between top-down task goals and bottom-up stimulus factors, providing an account of how these factors drive adaptive shifts in attention as a function of trial type.

Notwithstanding debate in the literature regarding how automatic and controlled channels interact (see Moors and De Houwer (2006) for a review on automaticity, and Cohen (2017) for a review on cognitive control), identifying changes in channel activation specified by Eqs. 3 and 4 with shifts in attentional resources provides a level of mechanistic specification that goes beyond the pulse function used in DMC. In particular, it is unclear whether automatic processing is affected by the increasing influence of the controlled channel (Hommel, 1993, 1994), or if it is actively suppressed (Ridderinkhof, 2002a). As discussed above, DMC’s pulse function is agnostic to mechanism—it describes the outcome of a change in processing without requiring specification of the underlying causal mechanism—and so the withdrawal of evidence outputted by the automatic channel can be interpreted as leakage (or passive decay), active suppression, or both (White et al., 2018). By contrast, in RDMC, attentional resources are actively shifted away from the automatic channel on incongruent trials with higher priority than on congruent trials, in line with the requirements of the task and the observer’s goal to respond accurately, modulated by the level of conflict among stimulus elements. Allowing for differences in attentional shifts across congruent and incongruent trials, when $k_c \ne k_i$, further underscores that shifting attention in RDMC is not a passive non-strategic process, but an outcome of the interaction of top-down goals with bottom-up congruency levels of the stimulus. The reallocation of processing resources requires no passive leakage of evidence, is inconsistent with notions of passively decaying channel activation, occurs without active suppression of the automatic channel, and does not involve the withdrawal of previously accumulated evidence. Instead, the speed with which goal-aligned shifts of attention are initiated depends on the presence of a bottom-up conflict signal that determines the urgency of enacting a shift.

More specifically, exogenous attention (e.g., the capture of attention by flanker arrows) and endogenous attention (e.g., responding to the target arrow) are the two different mechanisms instantiating the concepts of automatic and controlled channels in RDMC, respectively. We adopt an executive network perspective proposed by Botvinick et al. (2004) who extended the idea of focal attention to conflict resolution. They argue that the focal attention system is strongly related to executive control as the system can also produce top-down regulation in medial frontal cortex and anterior cingulate, which are often implicated in conflict resolution and conflict monitoring (Botvinick et al., 2001). Those two brain regions are activated on conflict trials in Stroop, Simon, and flanker tasks, hence conflict in these tasks share a common neurophysiological locus (Carter and van Veen, 2007). Further imaging evidence supports the occurrence of attention allocation in conflict tasks by suggesting that the dorsal anterior cingulate cortex is involved in attention allocation toward task-relevant stimuli, thus, limiting attention to distractors (Picard and Strick, 1996).

The output of the automatic and controlled channels is determined by the product of the relevant channel activation weight and a fixed channel-specific base drift rate parameter for automatic and controlled channels, $d_a$ and $d_c$, respectively. Note that the sign of the automatic base drift rate, $d_a$, is dependent on congruency condition, and is always positive on congruent trials and negative on incongruent trials. The controlled base drift rate, $d_c$, by contrast, is always positive. The channel-specific base drift rates describe the maximum levels of stimulus quality provided by task-irrelevant and task-relevant components of the stimulus (i.e., they index latent discriminability when attention is focused exclusively on either target or distractor information). We allow for $d_a \ne d_c$ to incorporate effects of the relative salience of target and distractor information into the model. For example, the greater number of distractor arrows in the flanker task may be especially potent in capturing attention, such that $d_a > d_c$. By incorporating the effects of discriminability and salience into a single value reflecting base drift rates, we implicitly combine these factors in our notation. A more complete expansion of factors influencing base drift rates would separate latent discriminability from salience for both targets and distractors, setting $d_a = d_c$ and allowing for independent estimation of salience multipliers for each term. In the current treatment, we avoid overcomplicating the notation with this information, but acknowledge that it may be desirable to separate salience from base drift rate under some circumstances. Theoretically, the base drift rate parameters capture our assumption that the encoded stimulus quality for different components of the stimulus should remain unchanged if the stimulus is fixed over the course of a trial. What can change dynamically is the degree to which information about each stimulus component enters the decision process, and this is determined by the channel activation weighting functions, $w_a(t)$ and $w_c(t)$. Following the same assumption as DMC, we assume that information from automatic and controlled channels are summed into a single combined evidence channel. In our model, the time-varying drift rate of the combined evidence channel is given by $v(t) = w_a(t)d_a + w_c(t)d_c$. Evidence accumulation in our model is therefore described by a Wiener process with drift rate *v*(*t*), $dX(t)=v(t)dt+\sigma dW(t)$, which can be expanded to match Eq. 2 with the exception that both automatic and controlled drift rates are time-dependent. Table 1 summarizes the parameters of RDMC.

**Table 1:** Model parameters

Symbol	Parameter
$A_{0}$	Initial activation level of the automatic channel
$k_c$	Attention shift rate for congruent trials
$k_i$	Attention shift rate for incongruent trials
$d_a$	Base drift rate for the automatic channel
$d_c$	Base drift rate for the controlled channel
*a*	Boundary separation
$T_{er}$	Mean of non-decision time
$s_t$	Range of non-decision time

In implementing RDMC, we follow the same conventional assumptions of the standard diffusion decision model (DDM) with regards to parameters not directly related to the behavior of the two processing channels (see Ratcliff 2013 for details about some of these parameters). We set the square root of the diffusion coefficient, $\sigma$, to 0.1. Non-decision time is assumed to be a uniform distribution with mean, $T_{er}$, and range, $s_t$.^12^ We depart from conventional DDM assumptions in the current presentation of RDMC by omitting between-trial variability in both drift rates and start-point. In the standard DDM, these parameters are responsible for controlling the relative speed of correct versus error responses. Drift variability produces errors slower than correct responses (Ratcliff, 1978). Start-point variability produces errors that are faster than correct responses (Ratcliff and Rouder, 1998). In the domain of typical conflict tasks, errors tend to be rare (e.g., $<5\%$ of total trials), and so for simplicity (and ease of parameter estimation), we do not include these parameters in our model. Of course, should the model be applied to a task where error rates are relatively higher and the ordering of correct and error RTs is a diagnostic feature of the data, expanding the model by incorporating these additional sources of parameter variability may be necessary.

The activation dynamics reviewed above lead to clear differences in the way distractor information influences decision-making in RDMC compared to the original DMC. Perhaps most importantly, the channel activation functions mean that evidence that is initially accumulated through the automatic channel persists through time rather than being eventually withdrawn from the decision mechanism. Critically, RDMC still predicts that the relative influence of this information diminishes over time because evidence from the controlled channel continues to accumulate over the course of a trial. Due to exponential decay of automatic channel activation, the rate at which evidence is outputted by the automatic channel decreases over time. If automatic channel activation reaches zero, the automatic channel ceases to output any new evidence to the decision mechanism. In this way, RDMC preserves the theoretical principle of a transition from automatic to controlled processing in conflict tasks.

Relating RDMC to other conflict models, we note that it shares some similarities with SSP, which assumes $k_{c}=k_{i}$ and $d_{a}=d_{c}$ in terms of the parameters of RDMC. That is, SSP assumes the same perceptual inputs of target and flanker stimulus^13^ and the same attention shrinking rate regardless of congruent and incongruent conditions. The mechanism of attention shrinking is conceptually similar to the idea of how attentional resources are withdrawn from the flankers and allocated to the target in RDMC, albeit expressed in spatial terms. The main difference between the two models is in the way changes in the spotlight (i.e., the distribution of attentional resources) are triggered by conflict. In SSP, attention always narrows in on the target, reflecting the top-down influence of task demands (i.e., identifying the target). In RDMC, the speed with which the “spotlight” is adjusted is modulated by the presence of bottom-up conflict, such that attention can shift more slowly on congruent trials.

In the next sections we show that RDMC achieves several key benchmarks including (1) the capacity to produce both positive- and negative-going delta plots, (2) strong parameter recovery properties, and (3) excellent fits to Ulrich et al. ’s (2015) joint choice and RT distribution data from both Simon and flanker tasks. These results demonstrate not just the viability of RDMC as a new general model of conflict processing, but also highlight how withdrawal of previously encoded evidence is not necessary for explaining negative-going delta plots.

### Parameter recovery

We now examine the parameter recovery properties of RDMC to assess the reliability with which its key parameters are estimated. We constructed synthetic data sets by sampling data-generating parameter values from ranges intended to resemble plausible values that might be found in fits to conflict data sets. Each individual parameter was randomly sampled from its own uniform distribution with an appropriate parameter range sufficient for generating both positive- and negative-going delta plots (see Table 2).

**Table 2:** Ranges of parameter values used in the parameter recovery study

$A_{0}$	$k_c$	$k_i$	$d_a$	$d_c$	*a*	$T_{er}$	$s_t$
0.6$-$0.9	0-80	20-80	0.3$-$0.7	0.4$-$0.8	0.07$-$0.15	300-370	70

We use the common chi-square method for fitting the diffusion model to data (Ratcliff and Tuerlinckx, 2002; White et al., 2018). In our applications, the model is simultaneously fitted to correct RT distribution data constrained by the error rate for each congruency condition. For each condition, the correct RT distribution data are sorted into six RT bins (formed by the.1,.3,.5,.7,.9 distribution quantiles). Error responses are assigned to one RT bin only since the error rate of conflict tasks is quite low (typically < 10%), and our goal is to explore RDMC’s parameter recovery properties under similar conditions to how the model might be applied to actual data sets. The expected frequency *E* in each RT bin is given by $E= {\sum }_{i=1}^{X}P_iN_i$ where $P_{i}$ is the proportion of responses and $N_i$ is the number of observations in each *i* RT bin. The chi-square goodness-of-fit is the sum of $\frac{(O-E)^2}{E}$ over the six correct response bins and one error response bin, where *O* stands for the observed frequency. Best-fitting parameter estimates are obtained by minimizing the chi-square value via the SIMPLEX routine (Nelder and Mead, 1965).

We simulated 40 data sets for three different numbers of observations per congruency condition (N=200, 500, 1000) to test how well parameters were recovered, given trial numbers similar to those found in some empirical studies (N=200). For fitting each synthetic data set, we randomly selected 10 starting points within $\pm 10\%$ of the generating parameter value to avoid local minima. The best-fitting parameter set was used to compare generating versus recovered parameter values. We quantify the quality of parameter recovery via the correlation (*r*) between the generating and recovered parameter values. Stronger correlations indicate better recovery results, and we use the same criteria as White et al. (2018) to assess the quality of parameter recovery: $.5<r<.75$ is considered fair, $.75<r<.9$ is good, and $r>.9$ is excellent.

**Fig. 5:**
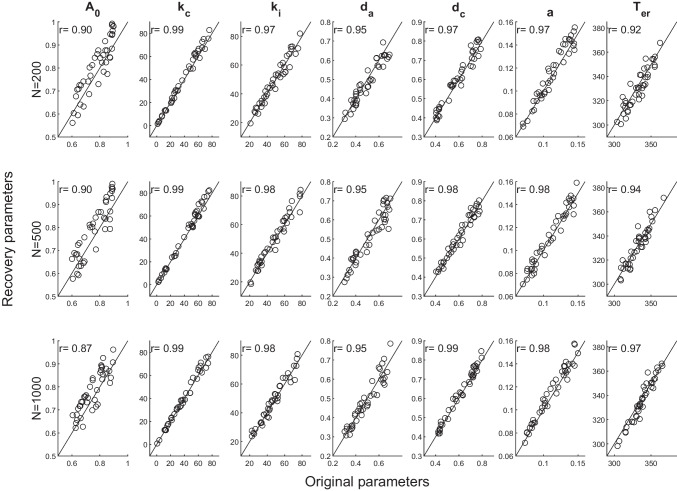
Parameter recovery for RDMC based on 40 simulated data sets. Each subplot shows the recovered parameter value plotted against the original data-generating parameter value. The correlation between the recovered and the original parameter values is used to quantify recovery, which is generally excellent

Figure 5 illustrates the correlation between the recovered parameters versus the generating parameters of RDMC as a function of N. In general, all model parameters show excellent recovery even at $N\ge 200$. The lone exception was near-excellent parameter recovery for the initial automatic channel activation parameter $A_0$, at $N\ge 1000$, $r=0.87$.

### Simulations of delta plots

A key challenge for theories of conflict processing is accounting for changes in the magnitude of congruency effects as a function of overall processing time (Pratte et al., 2010). Any general theory of conflict processing must be able to account for situations where the congruency effect increases over time as well those where the congruency effect diminishes over time. These different patterns are often observed in flanker and Simon tasks, respectively, producing positive- and negative-going delta plots. The following simulation exercise demonstrates how RDMC’s parameters affect the dynamics of conflict processing and, in turn, the slopes of delta plots. We show how the model can produce a range of delta plots by modulating the activation dynamics of processing in the automatic and controlled channels. We present the results of three simulations that vary channel activation dynamics via the $k_c$ parameter to manipulate the rate at which attention shifts away from the automatic channel on congruent trials. (See Table 3). For each parameter set, we simulated 4000 congruent and incongruent trials to approximate asymptotic model predictions from which we construct delta plots. Across the three parameter sets, the value of $k_c$ is varied to illustrate the role attentional shift dynamics have in determining the shape of the delta plot produced by the model.

**Table 3:** Data-generating parameters to illustrate the relationship between the $k_c$ parameter and the resulting delta plot predicted by the model

Figure							
	Parameters						
	$A_{0}$	$k_c$	$k_i$	$d_a$	$d_c$	*a*	$T_{er}$
6	0.8	2	30	0.4	0.6	0.094	300
7	0.8	10	30	0.4	0.6	0.094	300
8	0.8	20	30	0.4	0.6	0.094	300

For these simulations, non-decision time variability, $s_t=0$

**Fig. 6:**
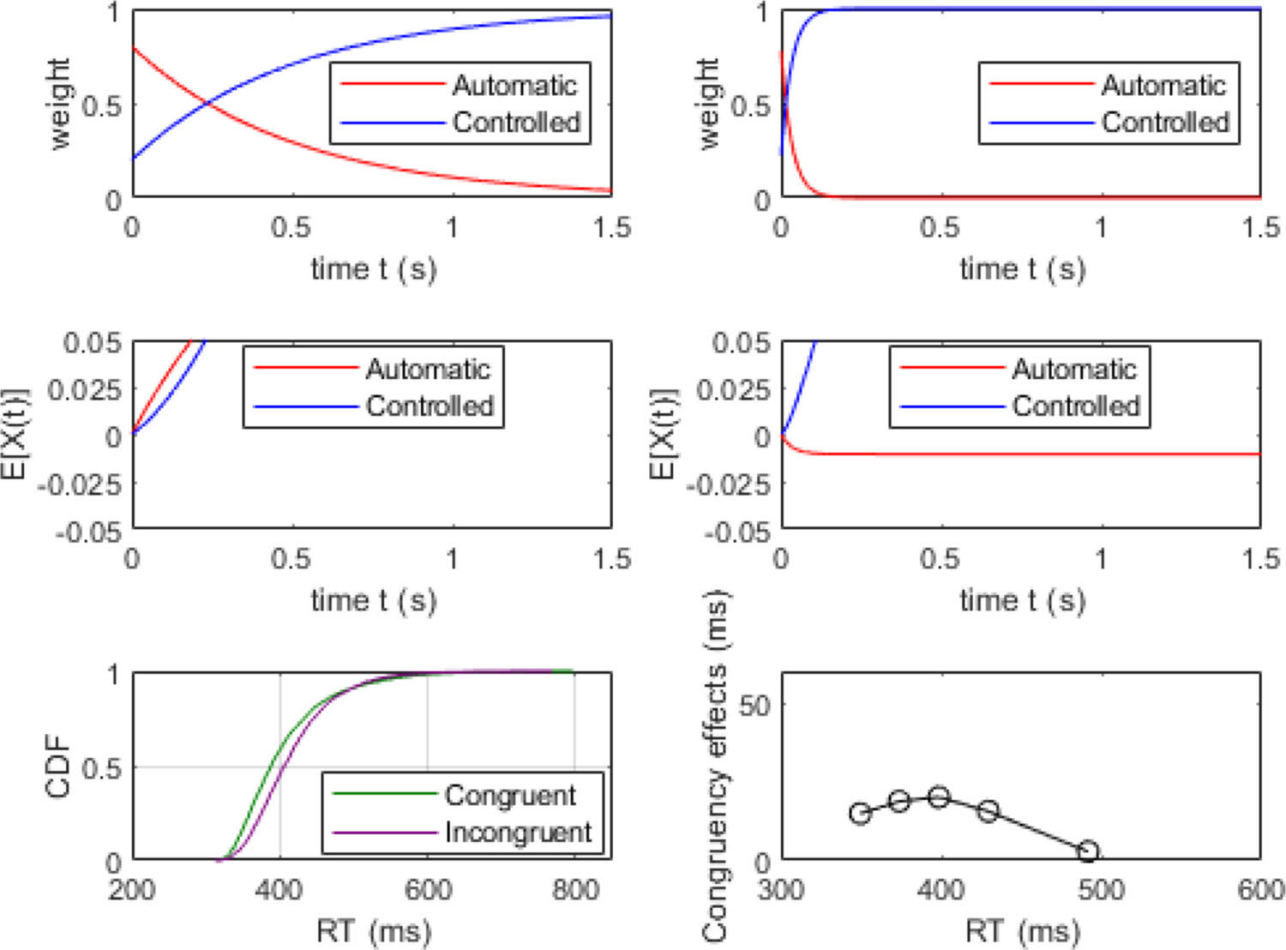
Simulation results of the parameter set in the top row of Table 3. The top two panels show channel activation dynamics on a congruent (*top left*) and an incongruent (*top right*) trial. The middle two panels show the expected evidence output from the automatic and controlled channels for congruent (*middle left*) and incongruent trials (*middle right*). *Red lines* represent automatic activation dynamics and *blue lines* controlled activation dynamics. Note that evidence accumulation is only drawn up to the limits of the two absorbing boundaries for illustrative purposes. The bottom two panels show cumulative RT distribution functions (*bottom left*) for congruent (*green line*) and incongruent trials (*purple line*) and the corresponding delta plot (*bottom right*)

Figure 6 illustrates the simulation results of slow attention shifting on congruent trials (i.e., data generated using the parameter set in the top row of Table 3). In this simulation, channel activation dynamics differ dramatically across different trial types, as attention shifts rapidly on incongruent trials (top right panel) but relatively slowly on congruent trials (top left panel). The resulting slow decay of automatic channel activation indicates an extended period of decision time where the information across both channels accumulates towards a common decision on congruent trials, as seen in the middle left panel of Fig. 6. Similarly, the fast attention shift reflects the time course of active interference produced by the distractor stimulus on incongruent trials. This early evidence works against responses being generated too quickly, as evidence accumulated by the automatic and controlled channels negate one another when combined. Once attention has shifted away from the automatic channel, evidence accumulation becomes dominated by the controlled channel (middle right panel of Fig. 6). Under these circumstances, RDMC produces a negative-going delta plot.

**Fig. 7:**
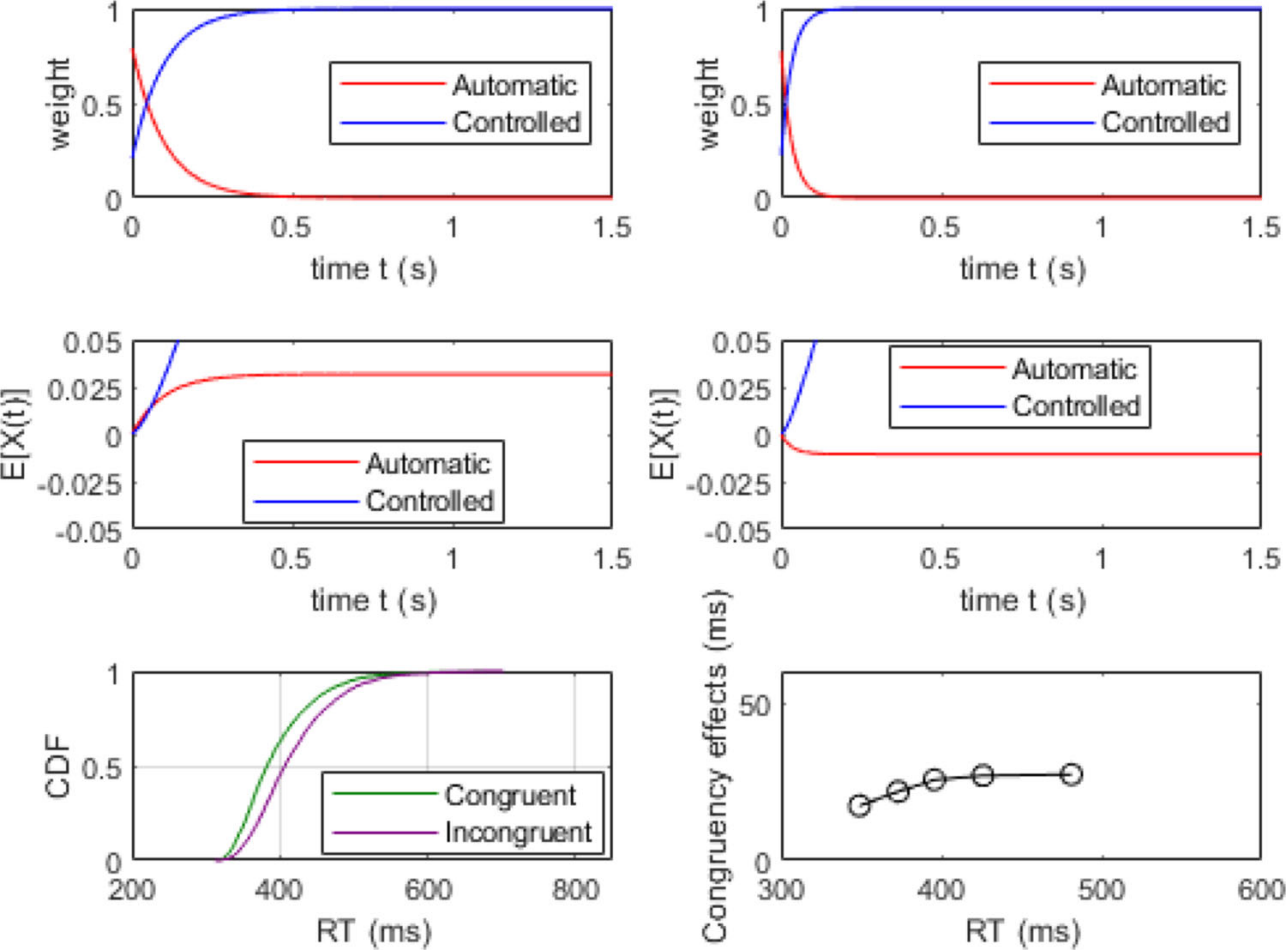
Simulation results of the parameter set from the middle row of Table 3. All plotting details are the same as Fig. 6

**Fig. 8:**
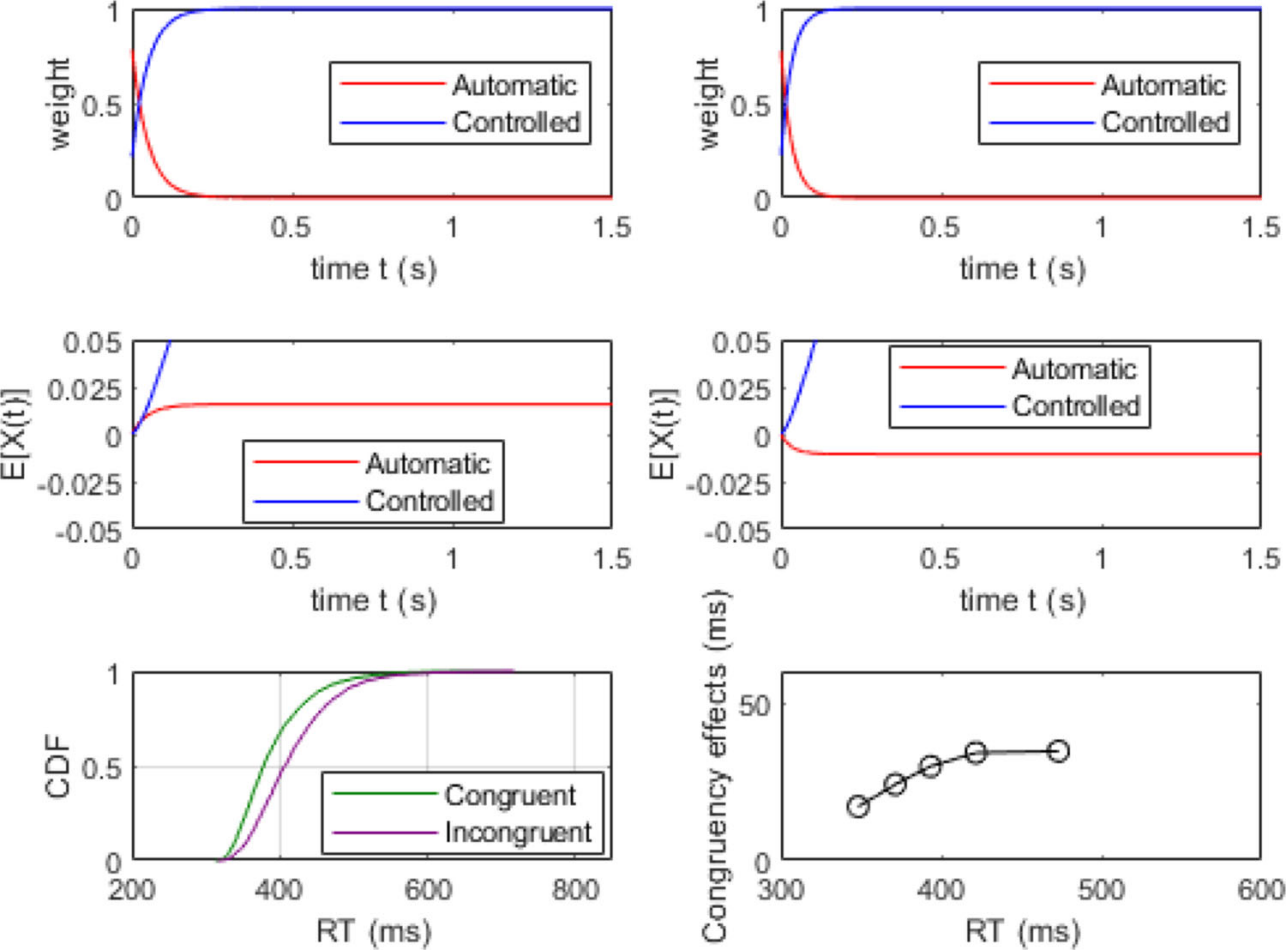
Simulation results of the parameter set from the bottom row of in Table 3. All plotting details are the same as Fig. 6

Figures 7 and 8 show the simulation results of the moderate and rapid attention shifts on congruent trials (i.e., data generated using the parameter sets in the middle and bottom rows of Table 3, respectively). In these simulations, attentional reallocation on congruent trials proceeds at a moderate rate (Fig. 7) and a fast rate (Fig. 8). As the attention shift becomes faster, there is less facilitation produced by the distractor stimulus. This means that the controlled channel comes to dominate the decision process more quickly, enabling a more consistent overall rate of evidence accumulation (see middle left panels of Figs. 7 and 8). In both of these cases, RDMC produces positive delta plots. The slopes of the delta plots depend on the rate of the attention shift. The faster the shift (i.e., the magnitude of $k_c$), the steeper positive slopes of the delta plots become (cf. the top left panel of Figs. 7 and 8).

**Fig. 9:**
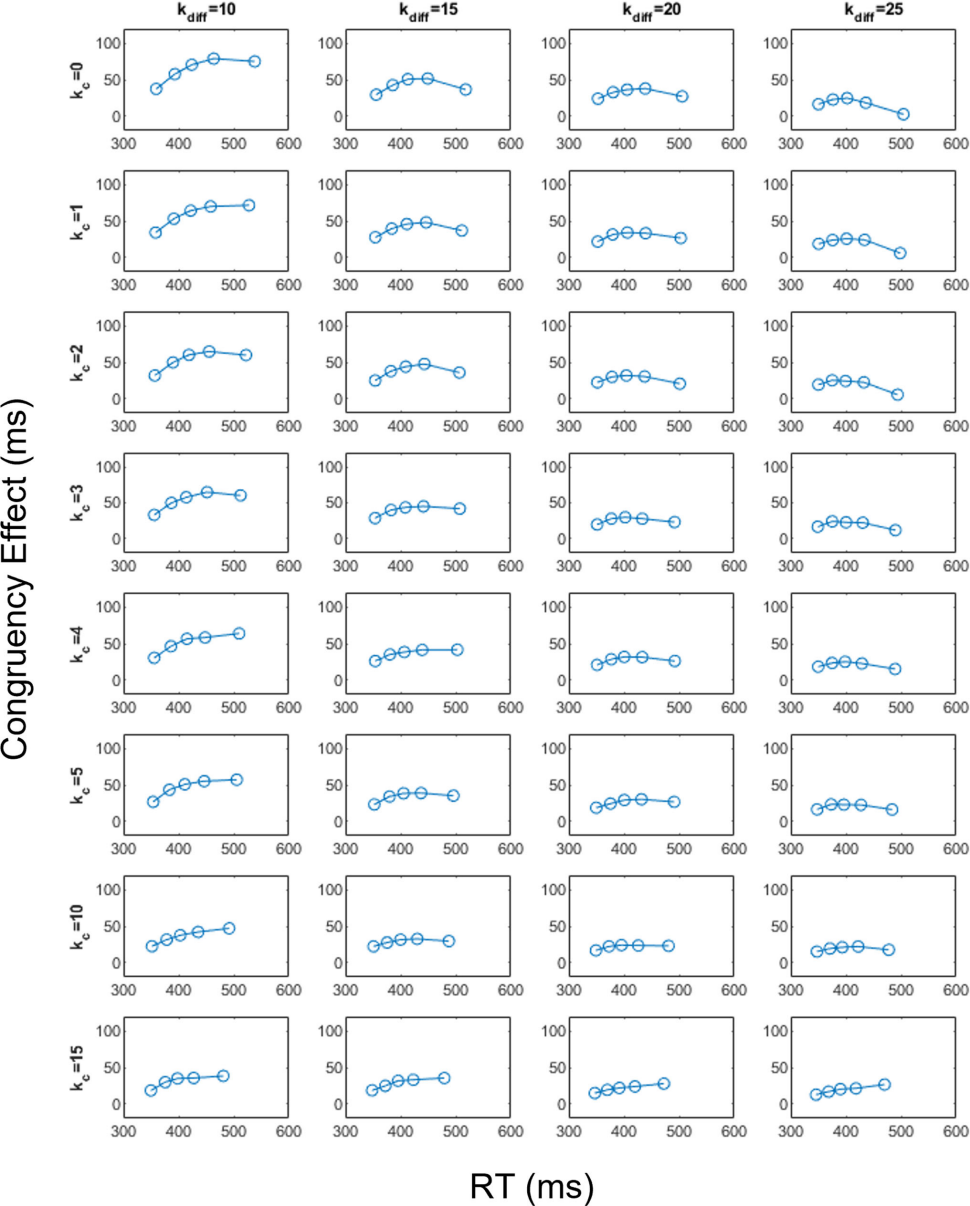
Delta plots produced by exploring a grid of parameter values for $k_c$ and the difference between $k_c$ and $k_i$ (i.e., denoted as $k_{diff}$). Each *dot* in each subplot represents the RT quantiles arranged from the fastest to the slowest (i.e., 0–20 %, 20–40%, 40–60%, 60–80%, and 80–100%)

To explore the conditions under which RDMC predicts negative-going delta plots in more detail, we created a grid of parameter values for both $k_c$ and the difference in attention shift rate across congruent and incongruent trials (i.e., $k_i-k_c$) and evaluated the slope of the resulting delta plot (see Fig. 9). The results of this exercise show that negative-going delta plots are predicted under conditions where (1) $k_c$ takes on a relatively small value, and (2) there is a large difference between $k_c$ and $k_i$. Suppose $k_c$ is small and difference between $k_c$ and $k_i$ is also small, that would imply $k_i$ is relatively small. In this case, on incongruent trials, there would be more fast errors along with slower correct responses due to the small $k_i$ (i.e., the influence of distractor information would persist for longer due to the slower attention shift rate). This would induce a large RT gap between congruent and incongruent CDFs. Therefore, the parameterization predicts a positive-going delta plot instead of a negative-going one.

### Modeling data with RDMC

Having demonstrated the strong parameter recovery properties of RDMC along with the model’s theoretical capacity to produce the range of delta plots that are observed in different conflict tasks, we now examine how well the model fits data from both Simon and flanker tasks. To facilitate comparability with the original DMC, we fit our RDMC to the group-averaged data from Ulrich et al. (2015). We also investigated fits to individual participant data from Ulrich et al. (2015). The data from the Ulrich et al. ’s (2015) study comprise both Simon and flanker task data that show clear differences in delta plots: negative-going for the Simon and positive-going for the flanker. While we focus on fitting the joint accuracy and RT distribution data for parameter estimation, we evaluate model performance by considering fits to these primary data in addition to other secondary features of the data that are emergent properties of the primary data. These include delta plots for the Simon and flanker tasks as well as the CAFs associated with each task. Because the latter two features of the data were not explicitly factored into parameter estimation, they serve as out-of-sample generalization tests.^14^

Ulrich et al. (2015) recruited 18 participants in total (two were omitted from the data analysis due to high error rates). Each participant completed the Simon and flanker tasks in a single experimental session, comprising eight blocks of 56 trials each, including 2 practice blocks. The Simon and flanker tasks were alternated across successive blocks (i.e., participants did not perform the same task across consecutive blocks.). Trial blocks were counterbalanced such that half of the participants started with the Simon task, and the other half started with the flanker task. Participants received feedback at the end of each trial, where the response was shorter than 150 ms (i.e., too fast), or longer than 1500 ms (i.e., too slow), or incorrect.

Data from the 16 participants—ignoring the two with high error rates—were used to compute group-averaged data via quantile averaging of the correct RT distribution data. Unlike Ulrich et al. (2015), we omit the two blocks of practice trials from the group-averaged and individual data. In all other respects, we follow Ulrich et al.’s treatment of the data and exclude trials with RTs shorter than 200 ms or longer than 1200 ms. We used the same chi-square approach for fitting the model to the Ulrich et al. (2015) data as we did for the parameter recovery study. We focused only on fitting the joint choice-RT distribution data for parameter estimation purposes. We then examined how well the model reproduces both delta plots and CAFs as an incidental byproduct of addressing these primary data. Because predictions from RDMC must be produced by simulation, we approximated asymptotic predictions from the model by simulating 8000 trials per condition to compare against data.

**Fig. 10:**
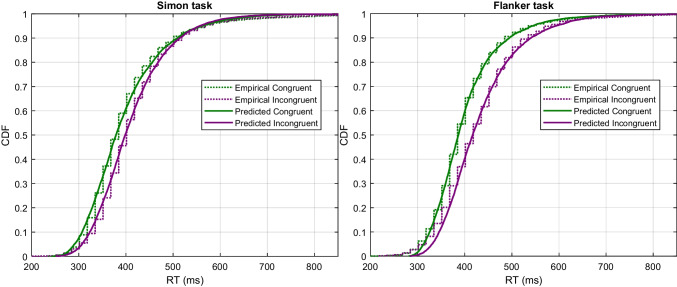
Observed and predicted CDFs of RT in Simon (*left panel*) and flanker (*right panel*) task. Each plot depicts congruent (*green*) and incongruent (*purple*) RTs separately

**Table 4:** Parameter estimates for the group-level and individual RDMC fits to Ulrich et al.’s (2015) Simon and flanker data

Task Type	Data Type	Parameters								
		$A_0$	$k_c$	$k_i$	$d_a$	$d_c$	*a*	$T_{er}$	$s_t$	$\chi ^2$
Simon task	Group	0.840	0.542	25.654	0.347	0.477	0.089	279.666	103.512	8.565
Flanker task	Group	0.789	42.477	93.039	0.822	0.373	0.081	312.476	94.832	11.716
Simon task	Individual	0.853	3.945	29.382	0.372	0.521	0.092	284.166	84.517	16.72
Flanker task	Individual	0.799	40.265	93.772	0.773	0.406	0.086	311.823	99.168	12.78

**Fig. 11:**
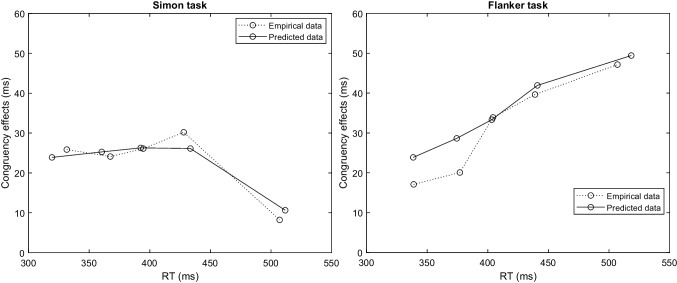
Empirical and predicted delta plots of Simon (*left panel*) and flanker task (*right panel*). The open plotting symbols connected by the dashed line represent the empirical.1,.3,.5,.7,.9 quantiles of the RT data. RDMC’s predictions are shown as *open symbols* connected by the *solid line*

**Fig. 12:**
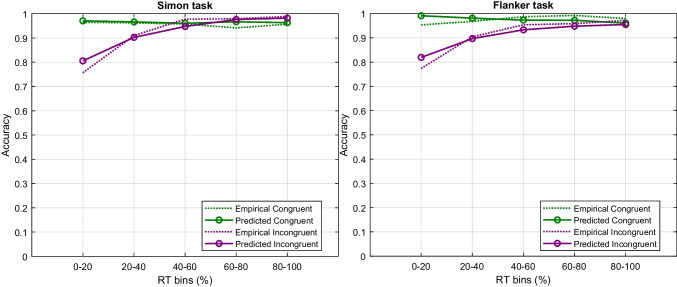
Empirical and predicted CAFs covering five equal-sized RT bins (i.e., 0–20%, 20–40%, 40–60%, 60–80%, 80–100%). Simon and flanker data are shown on the left and right panel, respectively, as open plotting symbols connected by the dashed line. RDMC’s predictions are shown by the *solid line*. Each plot depicts congruent (*green*) and incongruent (*purple*) RT separately

Figure 10 shows the predictions of RDMC against the group-averaged data from Ulrich et al. (2015). Data and fits for the Simon task are shown in the left panel, the corresponding information for the flanker task are shown in the right panel. Fits to the individual data are of comparable quality as the fit to the group-averaged data, and are shown in the Appendix. Notably, for the Simon data, several individuals show a negative congruency effect, where congruent RTs are slower than incongruent RTs. RDMC successfully captures these patterns in the individual data. We revisit the issue of negative congruency effects in the Discussion, where we report additional simulation results exploring their relationship with negative-going delta plots. The model provides an excellent fit to the primary choice-RT distribution data for both the Simon and flanker tasks. Table 4 shows the estimated parameters for the Simon and flanker task. As can be seen in Fig. 10, the predicted CDF of each congruency condition follows almost every pattern of the observed CDF. Importantly, the model shows close fits to changes in the difference in congruent and incongruent RTs over time: decreasing in the Simon task and increasing in the flanker task. The fact that the model closely captures these distinct features of the primary data from the Simon and flanker tasks is suggestive of its capacity to accurately predict the differences in delta plots observed across tasks. Figure 11 confirms this expectation, showing that the relevant delta plots are also captured by RDMC despite the model not being fitted directly to this secondary feature of the data. In particular, RDMC correctly generates a negative-going delta plot in the Simon task and a positive-going delta plot in flanker task. Although the accuracy rates across conflict tasks are generally very high (i.e., $\ge 90\%$), distinct patterns of accuracy as a function of binned RT are observed on congruent and incongruent trials of Simon and flanker tasks (Ulrich et al., 2015; Heitz, 2014). Figure 12 reveals the experimental and the model-predicted CAFs for the Simon and flanker tasks. In both tasks, accuracy is unconditionally high on congruent trials. On incongruent trials, however, accuracy rates are markedly lower when RTs are fast, and errors become far less frequent as processing time increases. Notwithstanding the differences in the time courses of the congruency effects captured by the model (as shown in the delta plots of Fig. 11), RDMC successfully accounts for the similar CAFs generated across tasks. Importantly, this secondary feature of the data did not directly inform parameter estimation, and so, like the delta plots predicted by RDMC, the CAF predictions can be considered as out-of-sample data that constitute a strong validation test.

**Fig. 13:**
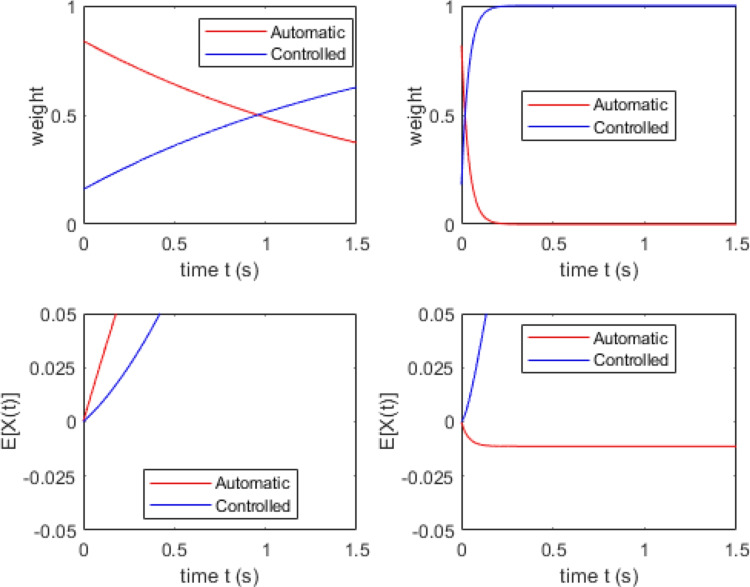
Channel activation dynamics predicted by RDMC for the Simon task. The top two panels show the predicted activation dynamics for congruent (*left*) and incongruent (*right*) conditions. The bottom two panels depict the model prediction of the expected evidence output of the automatic and controlled channels in congruent (*left*) and incongruent (*right*) conditions

**Fig. 14:**
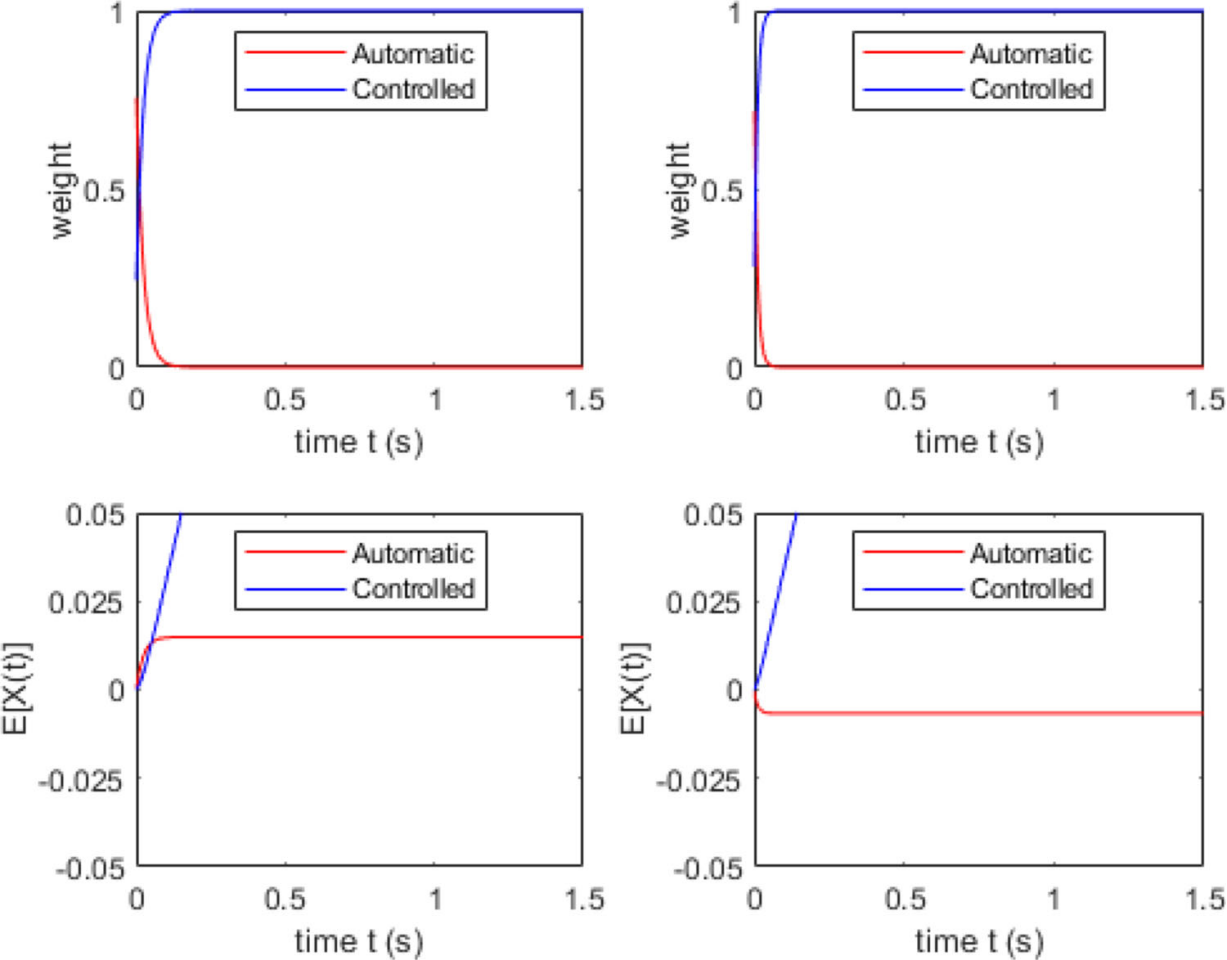
Channel activation dynamics predicted by RDMC for the flanker task. Plotting details are the same as Fig. 13

Best-fitting parameters estimates for both the group-averaged data as well as the average of the parameter estimates across individuals for the two tasks are shown in Table 4. Examination of the model parameters provides some insight into how the model accounts for performance in conflict tasks. The parameter estimates for the individual fits generally follow the patterns of the group-averaged estimated parameters. Indeed, the individual parameter estimates averaged across individuals are highly similar to the estimates based on fitting the group average, consistent with previous demonstrations fitting the standard diffusion model by Ratcliff et al. (2004, 2006). For both tasks, the initial activation value for the automatic channel is high (i.e., $A_0 > 0.75$), consistent with the idea that the task-irrelevant information—location for the Simon and peripheral stimuli in the flanker—dominates early processing. The high initial weight $A_0$ can be interpreted as reflecting the strong salience of the distractor information and its capacity for producing attention capture. Regardless of task, on incongruent trials, automatic channel activation rapidly decreases as attentional resources are reallocated from the automatic channel to the controlled channel. The allocation functions differ for congruent trials in a task-specific way: whereas attention is diverted away from task-irrelevant information for the flanker task (presumably controlled by a focusing of the attentional spotlight, as envisioned in the SSP model; White et al. , 2011), reallocation of resources is relatively slower in the Simon task. These differences highlight how the top-down strategic aspect of cognitive control interacts with bottom-up stimulus information. To avoid errors, attention must be reallocated to filter out task-irrelevant information on incongruent trials, but on congruent trials, this reallocation is not necessary to achieve accurate performance. Consequently, attention shifts in congruent conditions are less urgent to initiate. The more urgent attention shifts in the flanker task likely arise from the high salience of the distractor information, reflected in $d_a > d_c$ for the flanker, which differ from the Simon, where $d_a < d_c$. We conjecture that the highly salient distractor elements in the flanker provide a stronger bottom-up conflict signal that engages the attention shift mechanism more strongly than the conflict signal produced by location in the Simon.

Regarding the simulation exercise reported earlier, the slopes of delta plots were determined by how quickly attentional resources were reallocated on congruent trials (i.e., differences in the $k_c$ parameter). The fits to the Ulrich et al. (2015) data provide further support of these simulation results, showing how $k_c$ determines the slope of the predicted delta plot. Figures 13 and 14 illustrate the activation dynamics predicted by RDMC for congruent and incongruent trials for the Simon and flanker tasks, respectively. They are presented in the same way as in the earlier simulation exercise. Much like the simulated delta plots presented above, we see that RDMC, when fitted to Simon data featuring a negative-going delta plot, assumes channel activation dynamics that differ considerably across congruent and incongruent conditions. For congruent trials, there is relatively slow reallocation of processing resources across channels. For incongruent trials, there is rapid reallocation of resources away from the automatic channel and onto the controlled channel (see Fig. 13). Figure 14 shows that for the flanker data, distractor information stops accumulating at a very early stage of decision processing on both congruent and incongruent trials, reflecting unconditional distractor filtering. Overall, RDMC’s account of empirical positive- and negative-going delta plots aligns with the results of our simulation exercise: negative-going delta plots arise when distractor information is not rapidly filtered out on congruent trials.

## Discussion

The present study aimed to develop and test a new model of conflict data, RDMC, that implements different processing assumptions from the original DMC proposed by Ulrich et al. (2015). We examined the parameter recovery properties of RDMC and fitted the model to benchmark Simon and flanker data from Ulrich et al. (2015). We have shown that the new model, which enforces consistent representation of distractor information during processing, successfully accounts for all of the key benchmark data used to support the original DMC. Importantly, the processing assumptions of RDMC—being tied to the dynamics of attention reallocation—are mechanistically more clearly-defined than those underpinning the original DMC, while also simplifying, or removing ambiguity about, the relationship between fixed properties of the stimulus and the cognitive representation of those stimulus elements during processing. In adopting these assumptions, we are able to account for a broad range of conflict effect data within a framework where evidence is never withdrawn from a decision mechanism, inhibited, or leaked away. We show that negative-going delta plots can be produced in a theoretically defensible way, by purely feed-forward assumptions about processing. Once evidence is accrued by either the automatic or controlled channel, it persists until a response is made. Further, we showed that RDMC exhibited excellent parameter recovery with realistic experimental trial counts (N=200). The results of model fitting have shown that RDMC is able to account for the quantile RT data of the Simon and flanker tasks, incidentally predicting their delta plots (positive-going for the flanker task and negative-going for the Simon task) and CAFs.

RDMC closely fits the Simon and flanker data of Ulrich et al. (2015) in terms of the RT distributions, the corresponding delta plots, and the CAFs. The Simon data (i.e., negative-going delta plots) are incidentally predicted when distractor information is slowly filtered via gradual decay of automatic channel activation on congruent trials, but rapidly filtered on incongruent trials (i.e., when automatic channel activation decays rapidly). Referring to the flanker data, distractor information rapidly ceases to accumulate in both congruent and incongruent conditions, reflecting rapid shifts of attention for both trial types which is compatible with Servant and Logan (2019), suggesting similar attention focusing dynamics on congruent and incongruent trials. The speed of attention focusing is influenced by the interaction of top-down and bottom-up factors. Without a strong bottom-up conflict signal (i.e., congruent trials) driving rapid attention shifts, the activation of the top-down goal representation alone provides the impetus to shift attention (Folk and Remington, 1998). A question raised by our modeling analysis concerns across-task differences in the strength of the bottom-up conflict signal relative to the top-down goal influence of identifying the target. For the flanker task, the influence of the top-down goal may be amplified by the spatial arrangement of the flanking stimuli (i.e., resolving the details of the target may require focused attention; Strasburger , 2005). In contrast, for the Simon task, where the target is presented in isolation, such precise attentional focus may be unnecessary, or less vital, for achieving the goal of target identification. The simulation exercise and the model-fitting results suggest that the slopes of the delta plots are modulated by how long distractor processing remains active on congruent trials. Our modeling result also highlights that variation in delta plots may be understood in terms of how distractor information is processed on congruent trials instead of incongruent trials. RDMC allows for cognitive control dynamics—when and whether there is an attention shift that reallocates processing resources away from distractor information—to differ with regards to task and trial type, which allows for more fine-grained measurement of task performance (see also Evans & Servant, 2022).

In specifying attention shifting as the primary mechanism in RDMC, our model introduces different processing assumptions than the original DMC. Although this results in a comparable number of model parameters (i.e., both models used eight freely estimated parameters to fit the data of Ulrich et al., 2015, however, Ulrich et al. estimated the diffusion coefficient in a way that produces no effect on the resulting model predictions, rendering the effective number of free parameters in their original fits equal to seven; see Donkin et al., 2009, for discussion of the parameter scaling properties of sequential sampling models), there are potential concerns that the assumptions in RDMC might afford additional and unwarranted flexibility. To this concern, we respond that DMC is a mechanism-agnostic model, and is therefore conceptually more flexible, as the parameters governing the pulse function are fairly open to mechanistic interpretation. If DMC is shown to account for a particular data set, there is uncertainty about the theoretical mechanism that is implicated by the model’s success. We note that DMC was deliberately designed in this way to accommodate multiple (potentially competing) processing architectures. While this kind of conceptual flexibility has clear advantages—it identifies broad classes of theoretical frameworks or mechanisms that have the capacity to explain specific patterns of data—the downside of being unable to support specific processing mechanisms strongly limits theoretical inferences. RDMC’s commitment to an attention shifting mechanism is conceptually more constraining. Not only must the model explain how specific processing components interact with one another, but it must also show that these interactions are sufficient to reproduce key patterns in data. This increases the scope for falsification in that a failure of the model directly implicates a failure of an attention shifting account of data. We view this as a strength of our model, as it allows for detailed investigation of the viability of a class of processing assumptions for explaining a variety of conflict effects.

### Factors determining delta plots

Our investigation of RDMC also raises an intriguing question about whether we can control the shapes of delta plots by manipulating task design. Our model predicts a gradual slope change in delta plots by varying congruent activation dynamics controlled by the $k_c$ parameter. Hübner and Töbel (2019) show that negative-going delta plots—though not as steep as those found in Simon tasks—can be observed in a flanker task by varying the stimulus-onset asynchrony of flankers and the central target (i.e., when flankers are presented before the target). Instead of a general argument of stronger “inhibition” transitioning from a positive- to a negative-going delta plot predicted by Ridderinkhof (2002a, b) and the original DMC, our model proposes a new idea that attentional resources remain allocated to processing flankers on congruent trials or are withdrawn more slowly due to a weaker bottom-up conflict signal. More work is needed to explain why cognitive control is not always enacted on congruent trials. Effort and motivation could be associated with the allocation of control and willingness to pay the (cognitive) cost of enacting control (Kurzban et al., 2013; Botvinick and Braver, 2015). The idea can be tested by manipulating rewards/costs of correct/error responses or imposing a time-pressured conflict task. For instance, participants should be better able to filter out congruent and incongruent distractors when they receive rewards for doing so compared to those who are not rewarded. Some support for this idea has been provided by Padmala and Pessoa (2011) and Etzel et al. (2016), who employed a Stroop-like task to control incentives. Under these circumstances, we predict that attentional resources will be more likely to be reallocated from the automatic channel to the controlled channel in a reward-based Simon task. This leads to the expectation that a positive-going delta plot will be obtained if the incentive manipulation encourages unconditional shifts of attention, as found in our modeling results for the flanker task.

**Fig. 15:**
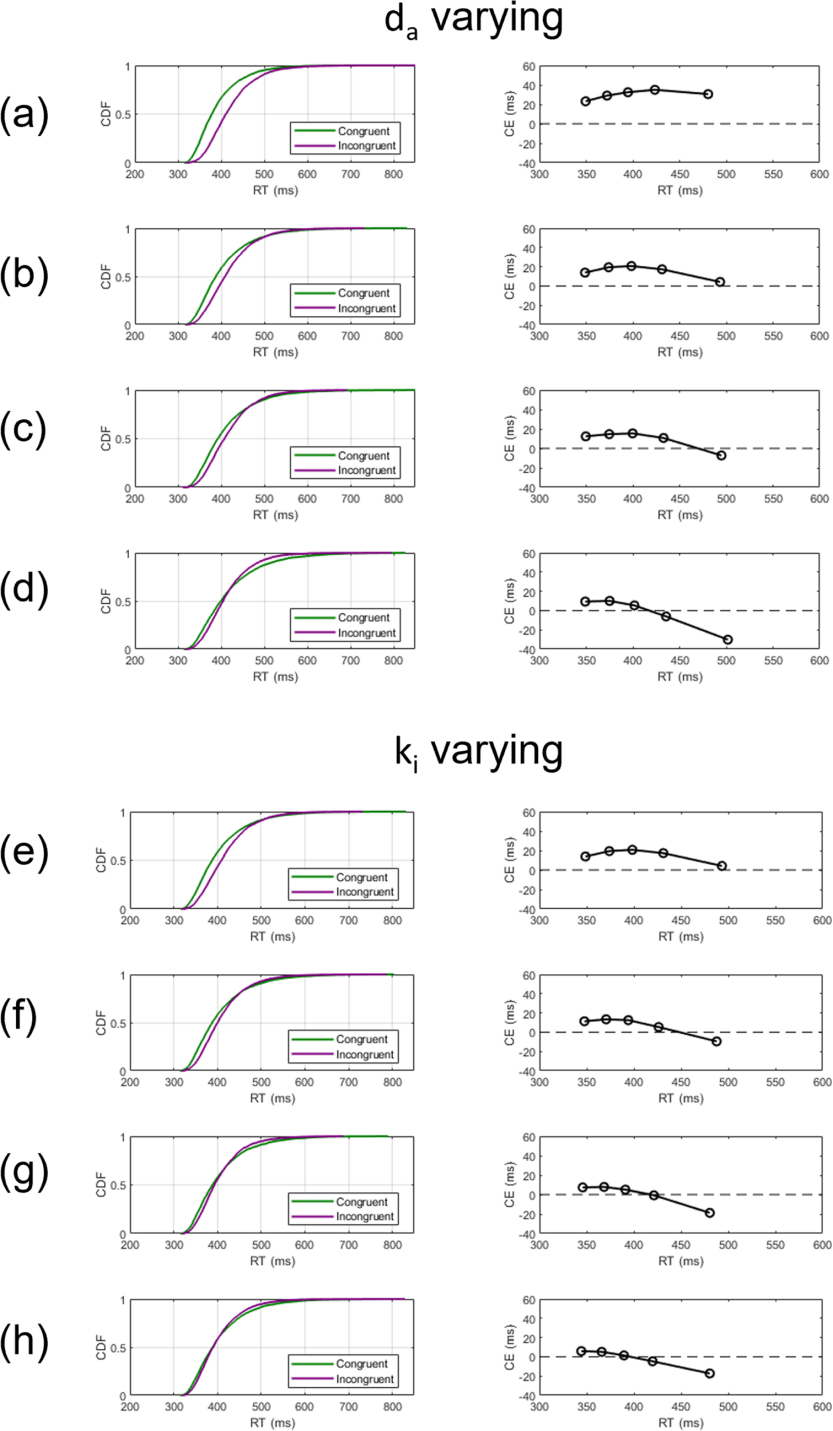
Simulated CDFs and delta plots of the parameter set from Table 5. Note that panels (b) and (e) are identical

### Asymmetrical facilitation and interference

Since the asymmetry of resource allocation on congruent and incongruent trials is essential for predicting the Simon data or any other conflict tasks with negative-going delta plots, we must reconsider the theoretical assumption of symmetrical processing dynamics—facilitation and interference—on congruent and incongruent trials in DMC. Facilitation and interference are two separate effects in the concept of executive function, but whether they are independent has been debated. Kornblum et al. (1990) assumes both effects are identical in congruent and incongruent trials as automatic and controlled processing modes are viewed as independent. However, previous literature, by either implementing neutral trials (e.g., Craft and Simon 1970; Eriksen and Eriksen 1974; Simon and Small 1969) or adopting a model-based approach (e.g., Evans and Servant 2022), has documented the imbalance of the facilitation and interference across conflict tasks. Specifically, an absence of facilitation on congruent trials has been observed in the presence of interference on incongruent trials in both Simon and flanker tasks. In RDMC, automatic and the controlled channel activations are not independent as decreasing automatic channel activation necessitates an increase in the controlled channel activation due to limited attentional capacity (Eqs. 3 and 4). Although distractor information may be identical on congruent and incongruent trials, attentional resources may not be fully reallocated on congruent trials. This is somewhat analogous to how responses are usually elicited before the attentional spotlight narrows completely to the central target on congruent flanker trials in SSP (White et al., 2011). Our findings suggest that the time course of enacting cognitive control over attentional resources depends on the interaction between the task-level response strategy adopted by the individual and bottom-up stimulus factors. According to RDMC, conflict is not the sole driver of attention shifts. The allocation of attentional resources is both sensitive to top-down (i.e., task goals) and bottom-up (i.e., salience of stimuli and the presence of conflict among stimulus elements) influences. To illustrate our findings, there is rapid attention shifting regardless of the presence of conflict in the flanker task—potentially reflecting a strategic policy of attentional control adopted by the individual and/or the necessity of attention to resolve fine visual detail about the target—whereas attention shifting appears more sensitive to trial-level congruency in the Simon task. The slow attention shifts observed on congruent trials in the Simon task might reflect a weaker stimulus-driven conflict signal to prompt shifting attentional resources across different processing channels.

Our finding that attention is slow to shift on congruent trials in the Simon task might seem to conflict with previous non-modeling studies that showed no facilitation effect in both Simon and flanker tasks (Craft and Simon, 1970; Eriksen and Eriksen, 1974; Simon and Small, 1969; Evans and Servant, 2022). The basic idea is that failing to quickly shift resources away from the automatic channel is tantamount to facilitating processing of the target on congruent trials. However, we argue facilitation effects are more nuanced than this, and that a model-based approach is essential for understanding them in full. RDMC disentangles the time course of channel switching from the quality of information carried by each channel naturally (i.e., the different functional roles of the *k* vs. *d* parameters). Yet facilitation can only be understood when considering both of these factors in combination (i.e., how base drift rate information interacts with the time course of attention-driven changes in channel activation). We found no facilitation effect in the Simon task since the base drift rates for automatic and controlled channels differed such that $d_a < d_c$ (see Table 4). It follows that complete and rapid reallocation of processing resources on congruent trials would produce a higher overall drift rate (facilitation) than the far slower and more gradual reallocation we observed (no facilitation). For the flanker task, we argue that despite $d_a > d_c$, facilitation remains absent because of the unconditional speed and completeness of resource reallocation across congruent and incongruent trials. For both congruent and incongruent trials, distractor processing is short-lived as attention resources are rapidly shifted away from the automatic channel. We argue that combining use of RDMC with novel experimental designs to specifically test facilitation is the most instructive way forward.

### Relation to other models and tasks

RDMC can produce positive- and negative-going delta plots by varying the attention shift rate on congruent trials, $k_c$, but it is not the only model that can account for different delta plots. Recently, Miller and Schwarz (2021) developed a descriptive race model based on the activation suppression framework proposed by Ridderinkhof (2002a, 2002b). This new race model can produce negative-going delta plots by adjusting the conditional probability of inhibition. The new race model assumes a discrete two-stage decision process, similar to DSTP (Hübner et al., 2010), instead of the idea of continuous attentional selection assumed by SSP (White et al., 2011) and RDMC. Future research will need to examine and compare the different properties of these models. For now, we suggest that a conceptual advantage of models like RDMC and SSP is that they are mechanistically more well-defined than the descriptive model of Miller and Schwarz (2021) that focuses on modeling the finishing times of different processing stages.

**Table 5:** Data-generating parameters to illustrate the relationship between the $d_a$ (panels (a), (b), (c), (d) of Fig. 15) and $k_i$ (panels (e), (f), (g), (h) of Fig. 15) parameters and the resulting negative congruency effects predicted by the model

Subplot							
	Parameters						
	$A_{0}$	$k_c$	$k_i$	$d_a$	$d_c$	*a*	$T_{er}$
a	0.8	2	30	0.5	0.6	0.094	300
b	0.8	2	30	0.4	0.6	0.094	300
c	0.8	2	30	0.35	0.6	0.094	300
d	0.8	2	30	0.3	0.6	0.094	300
e	0.8	2	30	0.4	0.6	0.094	300
f	0.8	2	40	0.4	0.6	0.094	300
g	0.8	2	50	0.4	0.6	0.094	300
h	0.8	2	60	0.4	0.6	0.094	300

Note that panels (b) and (e) are identical. For these simulations, non-decision time variability is set such that $s_t=0$

In addition to providing good fits to Ulrich et al. ’s (2015) Simon and flanker data, RDMC can be used to examine a broad range of conflict tasks. For example, negative-going delta plots are not exclusive to Simon data, but they are typically found in cuing tasks and masked priming tasks (Burle et al., 2005; Ellinghaus and Miller, 2018). Future studies should investigate how well the model can accommodate data from these different tasks, and if the explanations of negative-going delta plots in particular are consistent across them. Given our current examination of RDMC, we would expect to observe similar conflict dynamics to the Simon task, that is, relatively slow attentional shifts when target and distractor information is congruent compared with relatively faster attentional shifts when this information is incongruent.

Another issue to address is whether RDMC can fit negative congruency effects (i.e., RTs that are faster on incongruent than congruent trials) that are commonly seen in cuing, Simon, and priming tasks (see Burle et al. 2005; Kane et al. 1997; Marble and Proctor 2000; Schoeberl et al. 2019; Tipper Tipper (1985); Van Schie et al. 2008; Eimer and Schlaghecken 1998). Usually, these RT data show negative-going delta plots with negative congruency effects appearing at late RT quantiles (i.e., a crossover of congruent vs. incongruent RTs as a function of overall processing time). We ran a new set of simulations using the same procedure as the previous simulation of delta plots to show RDMC’s capability of producing negative congruency effects. With other parameters fixed, the negative congruency effect appears when the automatic base drift rate, $d_a$, becomes smaller (See Table 5 and panels (a), (b), (c), (d) of Fig. 15), or the attention shift rate for incongruent trials, $k_i$, increases (See Table 5 and panels (e), (f), (g), (h) of Fig. 15). These simulations—as well as the fit to some of the individuals in the Ulrich et al. (2015) data set—show that RDMC can handle negative congruency effects without issue. Adjusting either of these parameters generates congruent and incongruent CDFs that cross over in the middle to late RT quantiles, producing negative congruency effects in the tail quantiles of the RT distributions. Within the model, it appears that multiple parameters can potentially contribute to the negative congruency effect. The simulations varying $d_a$ imply weaker representations of the distractor information, thus, relatively weaker evidence produced by the automatic channel early on in the decision process. However, we view the latter simulations with $k_i$ varying as more plausible, as these indicate faster attention shifts on incongruent trials relative to congruent trials. Importantly, the attention shift rate on congruent trials is as slow as the rate associated with RDMC’s production of negative delta plots. This suggests that negative congruency effects might arise from the same processing dynamics that produce negative delta plots with positive congruency effects. This is in line with the current literature (Burle et al., 2014; Pratte, 2021), which has rarely shown the negative congruency effect in positive delta plots (i.e., the first two RT quantiles are always positive). We leave the question of which parameter is most important for explaining the effect to future research.

### Concluding remarks

We perceive RDMC as a useful general theoretical tool for investigating how conflict information is processed across different conflict tasks. Like other evidence accumulation models, RDMC highlights the importance of analyzing conflict data at the level of overall RT distributions. Our model showed that slopes of delta plots can be examined rigorously by fitting RT distribution data of congruent and incongruent trials. We believe that further adapting RDMC to study executive function will prove helpful to identify the psychological mechanisms of prominent effects. For example, the congruency effect is often found to be smaller for trials following an incongruent trial than for trials following a congruent trial (Gratton et al., 1992; Kerns et al., 2004; Stürmer et al., 2002), the congruency sequence effect or Gratton effect. Understanding how different components of processing are affected by such sequence effects is a challenge for current theories, but applying well-specified models like RDMC to these problems provides an opportunity to gain new insights into them (see Koob et al. , 2023 for a recent DMC-based analysis of the congruency sequence effect).

In sum, we present RDMC as a theoretical alternative to inhibition-based conflict models that only allows evidence strength to change monotonically. We showed that RDMC provides excellent fits to data from Simon and flanker tasks, and naturally generates appropriate delta plots and CAFs. The model provides a general explanation of negative-going delta plots based on the relative speed of reallocation of attentional resources across different trial types. Importantly, whether the model produces a positive- or negative-going delta plot depends on attentional dynamics when target and distractor information are congruent rather than incongruent. Slower resource reallocation on congruent trials was associated with negative-going delta plots. This feature of RDMC highlights the controlled (or at least non-obligatory) nature of attentional reallocation in conflict tasks, determined by interactions between top-down goals and bottom-up stimulus information. Our research highlights the importance of examining stimulus-driven factors in conflict tasks more closely, and opens the door to future research that seeks to understand the specific factors that determine when and whether people shift attentional resources in a way that depends on within-trial conflict.

## Figures and Tables

**Fig. 16:**
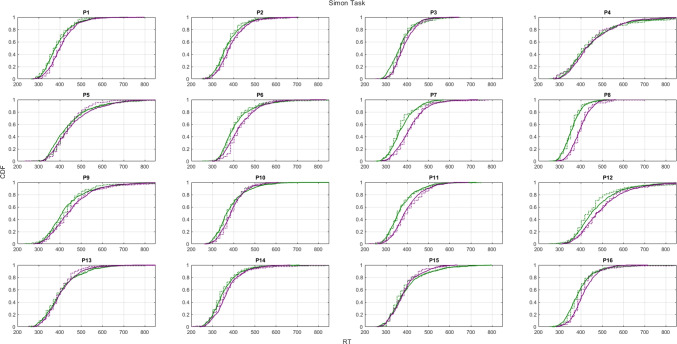
Observed and RDMC-predicted CDFs for individual RTs in the Simon task. Each panel depicts congruent (green) and incongruent (purple) RTs separately. Observed RT distributions are shown in dotted lines. Predicted RT distributions are shown in solid lines

**Fig. 17:**
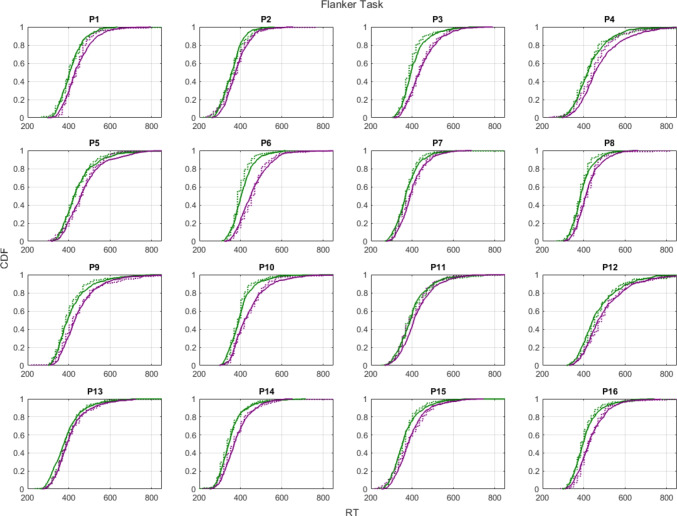
Observed and RDMC-predicted CDFs for individual RTs in the flanker task. Each panel depicts congruent (green) and incongruent (purple) RTs separately. Observed RT distributions are shown in dotted lines. Predicted RT distributions are shown in solid lines

## Appendix A: Model fits to individual subjects from Ulrich et al. (2015)

Below, we display individual fits of RDMC to the Simon and flanker RT data from Ulrich et al. (2015). The model-fitting procedure was the same used for the group-averaged data. Figures 16 and 17 show individual cumulative RT distributions for congruent and incongruent trials from the Simon and flanker tasks, respectively.

## Footnotes

1 Instead of letters, arrow stimuli are implemented in many flanker studies (White et al., 2011; Nieuwenhuis et al., 2006; Ridderinkhof et al., 2021; Tillman and Wiens, 2011).

2 Negative congruency effects are often found in Simon and cuing tasks with variable stimulus-onset-asynchrony between the task-irrelevant stimulus and subsequent target stimulus (Burle et al., 2005; Ellinghaus and Miller, 2018) We discuss negative congruency effects in more detail later in the article.

3 According to Schwarz and Miller (2012) and Miller and Schwarz (2021), negative-going delta plots are predicted when the mean RT of one condition, $RT_1$, is smaller than the mean of the other condition, $RT_2$, but the variance of $RT_1$ is greater than the variance of $RT_2$. The opposite assumptions hold for predicting positive-going delta plots. The misordering of moments in the aggregate RT data is readily produced within a mixture model framework comprising a fast process and a slow process (e.g., automatic and controlled processing), and importantly, can arise even when the means and variances of the pure component processes are ordered such that the fast process has both a smaller mean and variance than the slow process.

4 Ulrich et al. (2015) estimated the value of $\sigma$ freely from their data. Usually, $\sigma$ is fixed to either 0.1 or 1 by convention. This is because parameters in the diffusion model are identified at a ratio scale, changing the value of $\sigma$ by multiplying it by a constant means that all other parameters in the model that govern the evidence accumulation process can be scaled by the same constant to achieve the same predictions. Exclusions to this include non-decision time parameters, and in our model, the parameters that control the initial activation of the automatic channel and the two attention shift rates.

5 These dynamics are sometimes interpreted as arising from inhibition of automatic processing. We note that this interpretation rests on the assumption that inhibition not only prevents further accumulation of evidence through automatic processing, but also retroactively removes previously accumulated evidence from the decision mechanism. We fully agree with the idea that inhibition can halt subsequent processing, but are doubtful of views that inhibition can reverse or undo the effects of previously completed processing.

6 By *consistent* we mean that the encoded stimulus representation accords with a single response alternative during processing. Qualitative changes in drift rates are necessary in situations where stimulus information supports different response alternatives at different points in time (e.g., Holmes et al. 2016).

7 Alternatively, this could be achieved if inhibition prevents the automatic channel from accumulating evidence for only one of the two response alternatives.

8 A reviewer noted that negative congruency effects (i.e., RTs that are faster on incongruent than congruent trials) in masked priming tasks with relatively long stimulus onset asynchrony (SOA) between target and distractors (Burle et al., 2005; Eimer and Schlaghecken, 1998) can be interpreted as strong evidence for inhibition and the withdrawal of previously accumulated distractor evidence. However, the timescales associated with trials in these tasks require caution in interpretation, as it is likely that distractor information has been fully identified and processed before presentation of the target. With long SOAs, it is unclear whether a trial reflects a single, extended decision episode, or if it comprises multiple decision episodes. The dynamics associated with these cases are quite different, and so what the negative congruency effect arising from these studies implicates mechanistically is ambiguous (e.g., inhibition of early evidence accumulated within a single decision-making episode vs. inhibition of one response alternative across successive decision-making episodes). Further research is required to understand the mechanistic relationship between inhibition and evidence accumulation dynamics along with factors that determine the beginning and end of different decision-making episodes.

9 Technically, $A_0$ could take on any value between [0, 1], but we think values closer to 0 would only arise in quite unusual circumstances. For example, if a central target stimulus were to be shown prior to flanker stimuli in a flanker task. In such a case, it might not be unreasonable to assume that automatic channel activation could be completely shut down from the beginning of the trial, resulting in $A_0=0$.

10 Conceptions of attention in visuospatial tasks like the flanker are very naturally characterized as a spatiotemporal distribution of processing resources, sometimes characterized as a “zoom lens” (e.g., Eriksen and St. James 1986) to build on Posner et al. ’s (1980) original spotlight metaphor, and exemplified in conflict processing theories by the shrinking spotlight model (White et al., 2011). For other conflict tasks, like the Simon and Stroop, a more generalized conception of attention is required. Rather than thinking of attention in purely spatiotemporal terms, we follow the example from the category learning literature, and conceptualize attention as a distribution of processing resource dedicated to processing information across different stimulus dimensions (e.g., Nosofsky , 1986). Under this view, based on Shepard ’s (1980) classic work applying multidimensional scaling to characterize psychological representations, stimuli can be encoded based on a number of attribute dimensions, and processing resources can be distributed unevenly across those dimensions based on their task relevance (or salience). Stimuli in the visual version of the Simon task can be represented along two dimensions: an orientation dimension (corresponding to the target property) and a location dimension (an irrelevant distractor property). Traditional Stroop stimuli can similarly be encoded along dimensions capturing the (irrelevant) lexical properties of the stimulus and the (relevant) chromatic properties. In either case, attention shifting in our model assumes that processing resources initially allocated to the irrelevant dimension (e.g., due to bottom-up attentional capture) can be redirected to the relevant stimulus dimension. Our model instantiates the idea that attention mechanisms are responsible for allocating a limited pool of processing resources to restrict processing of task-irrelevant information and boost processing of task-relevant information.

11 ACC is sensitive to many forms of conflict. Regarding response conflict detection in Simon tasks, ACC has been highly sensitive to response conflict level (Bunge et al., 2002; van Veen et al., 2001; Weissman et al., 2003) and also it is strongly related to premotor, supplementary motor, and primary motor areas (Picard and Strick, 1996; Paus, 2001).

12 The variability of the non-decision time $s_t$ is required in the current model to blunt the leading edge of the predicted RT distributions to better match observed data. Empirical Simon and flanker data exhibit a similar gradual onset of RTs RN2 (Ulrich et al. , 2015). The same rationale motivated the introduction of non-decision time variability in the standard diffusion model (Ratcliff and Tuerlinckx, 2002). Without non-decision time variability, the model predicts a sharper onset of responding than is observed empirically.

13 White et al. (2011) allows different perceptual inputs of distractor stimuli (e.g., outer flankers and inner flankers) and target stimulus in their original model description. In that case, $d_a$ may not be equivalent to $d_c$ with respect to the parameters of RDMC.

14 Ulrich et al. (2015) fitted both RT distribution and CAF data simultaneously, incorporating an arbitrary scaling of accuracy units to RT to do so. Our fitting approach obviates the need for a conversion factor to address choice and RT.
